# Single‐Cell Transcriptomics and Metabolomics Reveal Glutamate Dehydrogenase as a Central Regulator of Nitrogen Metabolic Remodeling During Alkalinity Adaptation in Crustaceans

**DOI:** 10.1002/advs.76978

**Published:** 2026-08-05

**Authors:** Yiting Jin, Zhimin Lv, Chao Bian, Qibin Yang, Na Zhou, Jianguang Qin, Shengming Sun

**Affiliations:** ^1^ Key Laboratory of Exploration and Utilization of Aquatic Genetic Resources, Ministry of Education Shanghai Ocean University Shanghai China; ^2^ Laboratory of Aquatic Genomics, College of Life Sciences and Oceanography Shenzhen University Shenzhen China; ^3^ Tropical Aquaculture Research and Development Center, South China Sea Fisheries Research Institute Chinese Academy of Fishery Sciences Sanya China; ^4^ School of Pharmacy, Laboratory of Drug Discovery from Natural Resources and Industrialization, State Key Laboratory of Mechanism and Quality of Chinese Medicine Macau University of Science and Technology Taipa Macau China; ^5^ College of Science and Engineering Flinders University Adelaide Australia

**Keywords:** carbonate alkalinity, crustaceans, glutamate dehydrogenase, *Macrobrachium hainanense*, metabolic remodeling, nitrogen metabolism, single‐cell RNA sequencing

## Abstract

Alkaline environments disrupt ammonia excretion and challenge nitrogen metabolism in aquatic invertebrates, but the underlying cellular mechanisms remain unclear. To elucidate the adaptive mechanisms of crustaceans in highly alkaline environments, the responses of *Macrobrachium hainanense* to acute carbonate alkalinity stress are characterized using single‐cell RNA sequencing. High alkalinity inhibits normal ammonia excretion, leading to ammonia accumulation in the hemolymph, gill injury, mitochondrial dysfunction, and elevated oxidative stress. Marked heterogeneous remodeling occurs across distinct cell populations; pillar cells, nephrocytes, and semi‐granulocytes play primary roles in nitrogen metabolic regulation, acid‐base homeostasis, and immune defense, respectively. Further analyses identify glutamate dehydrogenase (GDH) as a key regulator of alkalinity adaptation. Inhibition of GDH significantly reduces alkaline tolerance, exacerbating tissue damage and metabolic disturbances while impairing ATP maintenance and inducing mitochondrial dysfunction under alkaline stress. Furthermore, GDH suppression inhibits urea metabolism while enhancing purine catabolism, indicating an adaptive shift in nitrogen metabolic strategy. This study provides a single‐cell resolution of crustacean alkalinity adaptation and identifies GDH‐mediated metabolic remodeling as a determinant of the environmental stress response. These findings offer theoretical insights into the regulatory mechanisms underlying stress adaptation in invertebrates.

## Graphical abstract

Single‐cell transcriptomics reveals the cellular response of Macrobrachium hainanense to carbonate alkalinity stress. Alkalinity stress impairs branchial ammonia excretion, induces mitochondrial dysfunction, and drives cell‐type‐specific changes in gills and hemocytes. Glutamate dehydrogenase sustains ATP homeostasis and coordinates nitrogen metabolic adaptation. Its suppression impairs urea metabolism, enhances purine catabolism, and reduces alkalinity tolerance, revealing an adaptive strategy for crustaceans in alkaline environments.

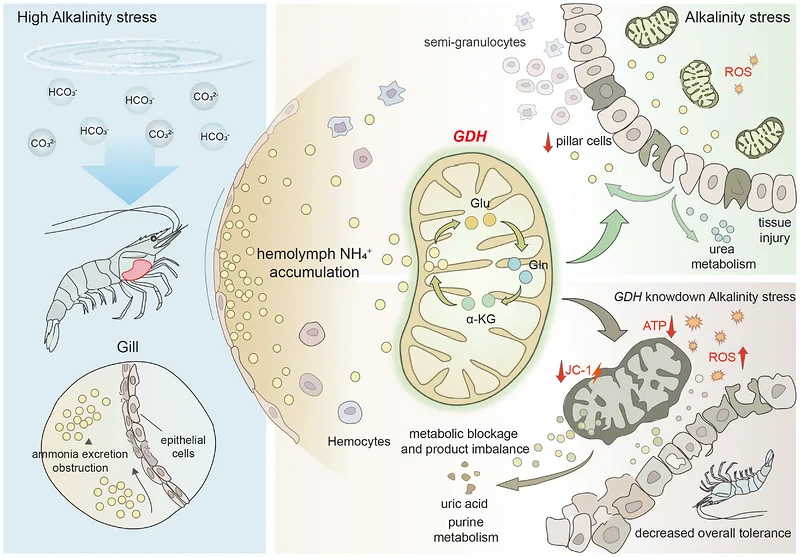

## 1 Introduction

Climate change represents one of the most pervasive drivers of global environmental change and is expected to profoundly impact aquatic ecosystems, including the expansion of global salinization [1, 2]. Saline–alkaline waters are typically characterized by high alkalinity, elevated pH, and ionic imbalance, rendering them unsuitable for agriculture and drinking, while simultaneously imposing severe physiological stress on aquatic organisms [3, 4]. With the increasing global demand for aquatic food resources, the development of aquaculture in saline–alkaline environments has emerged as a critical strategy for sustainable food production [5, 6]. Therefore, improving the utilization efficiency and economic value of saline–alkaline water resources requires a comprehensive understanding of the stress responses induced by salinization in aquatic animals.

In fish, adaptation to high alkalinity is a well‐characterized physiological process that begins with a reduction in H^+^ concentration, which limits the conversion of toxic ammonia nitrogen (NH_3_) into the less toxic ammonium ion (NH_4_^+^), thereby impairing branchial ammonia excretion and promoting internal ammonia accumulation [7, 8]. Under alkaline stress, fish mitigate this challenge by detoxifying excess ammonia through glutamine synthesis and, in some highly adapted species, by enhancing urea production via the ornithine–urea cycle, as observed in *Oreochromis alcalicus grahami* [9, 10, 11]. In contrast, in crustaceans, another group of economically important aquaculture species, the cellular responses to alkalinity stress and the underlying molecular mechanisms remain poorly understood. Although previous studies have examined alkalinity tolerance ranges and associated physiological changes in crustaceans, current evidence indicates that ammonia excretion primarily occurs across the gill epithelium and is driven by ion and acid‐base gradients maintained by Na^+^/K^+^‐ATPase, V‐type H^+^‐ATPase, and ammonia transport systems such as Rh/Rh‐like proteins [12, 13, 14]. Because ammonia is highly cytotoxic and can induce oxidative damage or even mortality, elevated environmental alkalinity increases the proportion of non‐ionic ammonia in the water, thereby promoting its accumulation within the organism and exacerbating toxicity and physiological disturbance [15, 16, 17, 18]. Therefore, maintaining efficient ammonia excretion is essential for crustaceans to adapt to saline–alkaline stress and preserve internal homeostasis [15, 19]. High alkalinity is known to impair nitrogenous waste excretion in crustaceans and can induce metabolic alkalosis. As the primary interface between hemolymph and the external aquatic environment, the gills function not only as the main site of ammonia excretion but also as a key organ for respiration, osmoregulation, and acid‐base balance [19, 20]. Although the antennal gland is recognized for its role in ion reabsorption and osmoregulation, the gill serves as the primary interface for ammonia excretion, ion transport, and acid‐base regulation in aquatic crustaceans [21]. In the open circulatory system of crustaceans, hemocytes and gill‐mediated transport processes are central to the regulation of acid‐base balance and ammonia homeostasis. In addition, hemocytes act as major immune effector cells, producing and releasing humoral factors and participating in stress responses [19, 22]. Recent studies have shown that carbonate alkalinity stress induces oxidative damage in gill tissues and disrupts ion transport processes, accompanied by increased ammonia levels in the hemolymph [23, 24]. However, the mechanisms by which crustaceans perceive and respond to alkalinity stress signals, as well as the specific cell types, genes, and signaling pathways involved across multiple tissues, remain largely unresolved.

To overcome the limitations of traditional RNA sequencing in resolving cell‐specific gene expression, single‐cell RNA sequencing (scRNA‐seq) has emerged as a powerful approach for investigating heterogeneous responses of aquatic organisms to environmental stimuli, enabling the identification of novel cell types and the reconstruction of cell lineage trajectories. ScRNA‐seq has been widely applied to characterize cellular states and functional responses in crustaceans under various stress conditions, including studies in Pacific white shrimp *Litopenaeus vannamei*, kuruma prawn *Marsupenaeus japonicus*, giant freshwater prawn *Macrobrachium rosenbergii*, and black tiger shrimp *Penaeus monodon* [25, 26, 27, 28]. The cell‐type‐specific markers identified from these single‐cell atlases provide valuable resources for advancing crustacean biology. Although scRNA‐seq has offered important insights into the cellular effects of alkalinity stress within a single tissue in ridgetail white shrimp *Exopalaemon carinicauda*, a comprehensive understanding of tissue‐specific heterogeneous responses and their coordinated regulatory networks across multiple tissues remains urgently needed in crustaceans [29].

In this study, the estuarine prawn *Macrobrachium hainanense* was selected as the model species because of its relatively high tolerance to alkaline conditions compared with other freshwater prawns, as well as its natural distribution in estuarine and coastal river systems of southern China [30, 31]. These characteristics make it a promising candidate for aquaculture in saline–alkaline water resources. Building on a previously established high‐quality genome, scRNA‐seq and metabolomic profiling were integrated to investigate cellular responses in gill and hemolymph tissues under alkalinity stress and to identify key ion‐transporting cell types involved in acid‐base regulation in a cell‐type‐specific manner [31]. Analysis of cell‐type‐specific differentially expressed genes (DEGs) and co‐expression networks revealed that alkalinity stress specifically induces the expression of glutamate dehydrogenase (*GDH*) in epithelial cells. Further investigation elucidates the role of GDH in alleviating ammonia toxicity and regulating nitrogen metabolism, thereby uncovering key pathways that maintain nitrogen homeostasis under alkaline conditions. These findings provide a mechanistic framework for understanding the adaptive strategies of aquatic invertebrates in response to alkalinity stress.

## 2 Results

### 2.1 Carbonate Alkalinity Stress Impairs Gill and Antennal Gland Structure and Disturbs Ammonia Metabolism in *M. hainanense*

Under carbonate alkalinity stress, both the gills and antennal glands of *M. hainanense* exhibited varying degrees of structural damage. In the control group, the gill architecture was intact, with well‐organized filaments and normal secondary lamellae morphology. After 96 h of alkaline exposure, the gill filaments became wrinkled and deformed, secondary lamellae showed hypertrophy and swelling, cellular organization was disrupted, and extensive apoptosis occurred along the gill axis (Figure 1A, E, F (a)). Concurrently, ultrastructural observations revealed pronounced vacuolization, severe microvillar damage, loss of mitochondrial double‐membrane integrity, and disruption of membrane structures (Figure 1C). In the antennal gland, the lumen was enlarged, inter‐luminal connections became looser and less compact, microvilli were damaged, and some brush border cells detached, accompanied by an increased number of apoptotic cells (Figure 1B,E,F(b)). Transmission electron microscopy (TEM) further showed that mitochondrial cristae were disrupted, although the overall organelle structure remained relatively preserved (Figure 1D).

**FIGURE 1:**
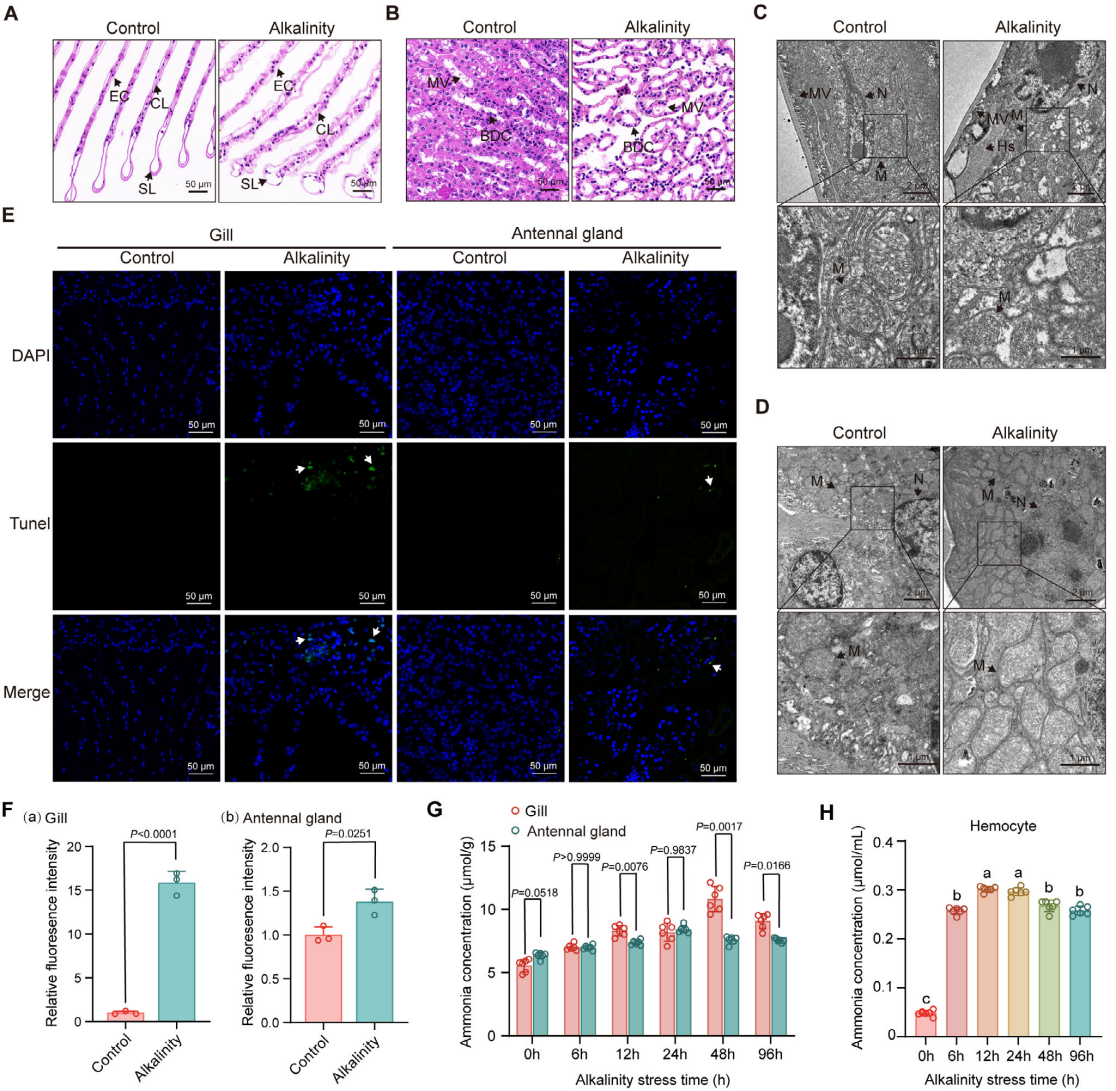
Histological alterations and ammonia metabolism analyses of the gill and antennal gland under carbonate alkalinity stress. (A, B) HE staining of the gill and antennal gland. (C, D) TEM images of the gill and antennal gland; boxed regions are shown at higher magnification below. (E) TUNEL staining of the gill and antennal gland where nuclei are stained with DAPI (blue) and apoptotic signals are shown in green; arrows indicate TUNEL‐positive cells. (F) Quantification of TUNEL fluorescence intensity. (G) Ammonia concentrations in the gills and antennal glands at different time points during alkalinity stress. Corresponding *P* values are indicated in the figure. (H) Changes in ammonia concentration in hemocytes over time during alkalinity stress, with different lowercase letters indicating significant differences among time points (*P* < 0.05). Abbreviations: EC, epithelial cell; CL, capillary lumen; SL, secondary lamella; Hs, hemolymph sinus; M, mitochondrion; MV, microvilli; N, nucleus; BDC, brush‐border cell. Scale bars: A, C, E, 50 µm; B, D (upper panels), 2 µm; B, D (lower panels), 1 µm.

To evaluate the effects of alkalinity stress on ammonia nitrogen metabolism in *M. hainanense*, the concentrations of ammonia and urea were measured in key metabolic organs, including the gills, antennal glands, and hemocytes. Under alkaline conditions, ammonia levels in hemocytes were consistently higher than those in the control group throughout the 6–96 h exposure period (Figure 1H). In both the gills and antennal glands, ammonium ions accumulated to varying extents, with concentrations showing an initial increase followed by a gradual decline over time. Notably, ammonia levels in the gills were significantly higher than those in the antennal glands at 12, 48, and 96 h (Figure 1G). Meanwhile, ammonia concentrations in the culture water were monitored throughout the experiment. Ammonia concentrations increased significantly at 6 h compared with the CK and 0 h groups and remained stable from 6 to 96 h (Figure S1C), indicating that waterborne ammonia levels were relatively constant during the subsequent alkalinity exposure. Urea levels in the hemolymph increased significantly at 12 h, then gradually declined, and were significantly lower at 96 h compared with baseline (Figure S1A). In the gills, urea content increased markedly at 6 h and subsequently decreased, yet remained higher at 96 h than at 0 h. In contrast, urea levels in the antennal glands increased steadily from 6 to 24 h before returning to baseline at 48 and 96 h, with no significant difference relative to 0 h (Figure S1B). These results indicate that alkalinity stress disrupts normal ammonium metabolism while promoting urea‐associated pathways, with the gills likely serving as a central organ in the regulation of ammonia nitrogen metabolism.

### 2.2 Identification of Cell Types in the Gills and Hemocytes and Their Heterogeneous Responses to Alkaline Stress

To characterize the cellular composition and heterogeneous responses of gill and hemocyte populations in *M. hainanense* under alkaline stress, scRNA‐seq was employed to generate single‐cell transcriptomic profiles. This approach enabled the investigation of key cellular mechanisms at single‐cell resolution (Figure 2A). Following quality control and the removal of low‐quality cells, a total of 48,928 gill cells and 43,339 hemocytes were retained for analysis. In gill cells, the median UMI count and the number of detected genes per cell were 1,515 and 785, respectively, whereas in hemocytes, these values were 2,081 and 1,034 (Table S1). Dimensionality reduction and visualization using Uniform Manifold Approximation and Projection (UMAP) and t‐distributed stochastic neighbor embedding (t‐SNE) identified 14 and 12 distinct clusters in gill cells and hemocytes, respectively (Figure S2). Cell types were subsequently annotated based on established marker genes reported in crustaceans, fish, and mammals. In the gills, nine cell types were identified, including three ionocyte subtypes, pillar cells (expressing *V‐ATPase*, *KCNJ12*, *ABCC5*, *GLUT1*, and *NHE1*), septal cells (expressing *ATP1B2*), and chloride cells (expressing *CA*), as well as nephrocytes (expressing *CUBN*, *Nephrin‐like*, and *LASP1*), endothelial cells (expressing *LPHN2*, *ANGPT2*, and *NOSTRIN*), pericytes (expressing *VEGFR1* and *ITGA8*), mucous cells (expressing *MUC5AC‐like* and *MUC17‐like*), neuroepithelial cells (expressing *AChe*, *KIF5*, *NGF*, and *DLX2*), and gill‐associated hemocytes (expressing *α2M*, *ALF*, and *proPO*) (Figure 2F; Figure S2). In the hemolymph, five distinct hemocyte populations were identified, including precursor hemocytes (expressing *HMGA1*, *IGFBP4*), differentiating hemocytes (expressing *TGase*, *histone H1‐delta*, *TUBA3*, and *SUV39H2‐like*), semi‐granulocytes (expressing *RAB11FIP4* and *β‐arrestin 1*), granulocytes (expressing *proPO* and *hemocytin‐like*), and specific granule‐containing hemocytes (expressing *glycine‐rich crustin 2*, *TUBB1*, and *PPAF*) (Figure 2G; Figure S2).

**FIGURE 2:**
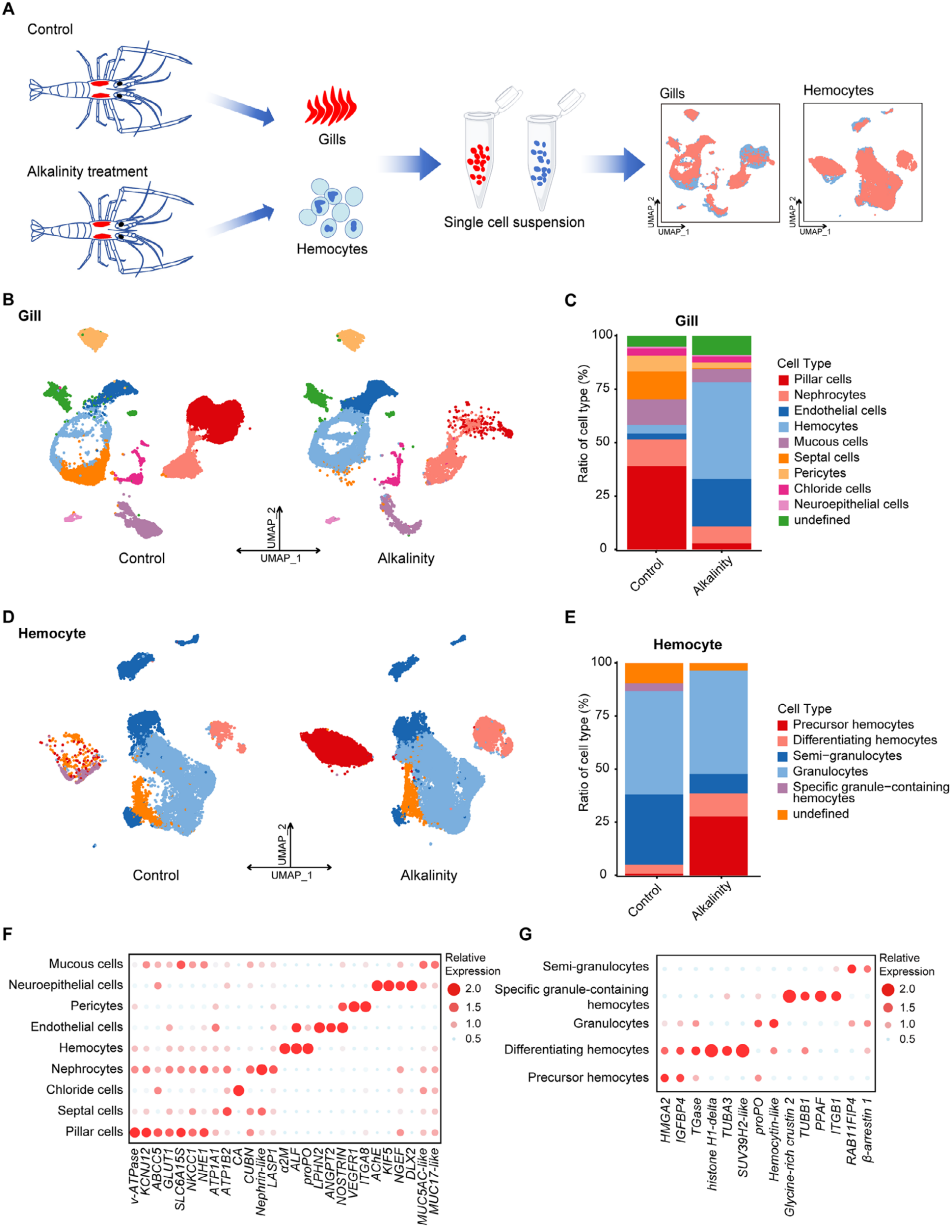
Single‐cell transcriptomic atlas of the gill and hemocytes under alkalinity stress. (A) Schematic overview of the experimental design and scRNA‐seq workflow. (B) UMAP visualization of gill cells from the control and alkalinity groups, showing the distribution of annotated cell populations. (C) Relative proportions of gill cell types in the control and alkalinity groups. (D) UMAP visualization of hemocytes from the control and alkalinity groups. (E) Relative proportions of hemocyte subtypes in the control and alkalinity groups. (F, G) Dot plots showing representative marker genes used for the annotation of gill cells and hemocytes.

Under acute alkalinity stress, both gill cells and hemocytes exhibited pronounced heterogeneous responses (Figure 2B–E). In the control group, the gill cell population was predominantly composed of pillar cells (38.99%), followed by septal cells (13.01%), nephrocytes (12.63%), and mucous cells (11.95%). In contrast, alkalinity stress markedly altered this composition, with the proportion of pillar cells sharply decreasing to 2.87%, indicating that this cell type is highly sensitive to alkaline conditions and may play a critical role in the stress response. Concurrently, the proportions of hemocytes and endothelial cells increased substantially, whereas septal cells and pericytes, in addition to pillar cells, showed notable reductions (Table S2). In the hemolymph, granulocytes (48.66%) and semi‐granulocytes (33.16%) were the dominant cell types under control conditions. Following alkalinity stress, the proportion of precursor hemocytes increased dramatically from 0.06% to 27.83%, and differentiating hemocytes also rose to 10.72%, while semi‐granulocytes decreased significantly from 33.16% to 9.19% (Table S2). These findings indicate extensive remodeling of both gill and hemocyte populations in response to alkaline stress.

Cell–cell communication analysis predicted pericytes and endothelial cells as major interaction hubs within the gill, with strong potential communication between these populations and additional interactions involving hemocytes (Figure S3A). Molecular docking of the representative ligand–receptor pairs RGMb–NEO1 and IL1RAPL1–PTPRF identified potential binding sites and intermolecular contacts, further supporting these predicted interactions (Figure S3B). These findings suggest that pericytes and endothelial cells coordinate local cellular responses within the gill while potentially mediating communication between gill cell populations and hemocyte‐associated immune activity. Subsequently, KEGG enrichment analysis was performed on DEGs from key cell clusters associated with the alkaline stress response (Figure S4). The results revealed pronounced functional specialization among different cell types under stress conditions. In the gills, DEGs in pillar cells were primarily enriched in pathways such as “Insulin secretion”, “Nitrogen metabolism”, and “ABC transporters”, highlighting their roles in ion and solute transport as well as nitrogen metabolic regulation. Nephrocytes and septal cells showed significant enrichment in “Glycolysis/Gluconeogenesis”, “Collecting duct acid secretion”, and “Phagosome”, suggesting key functions in energy metabolism, maintenance of acid‐base homeostasis, and cellular defense. Pericytes were enriched in the “MAPK signaling”, “regulation of the actin cytoskeleton”, and “C‐type lectin receptor signaling” pathways, whereas endothelial cells preferentially enriched the “Ras”, “PI3K–Akt”, and “VEGF signaling” pathways, suggesting complementary roles in coordinating local immune responses, maintaining tissue integrity, and promoting stress adaptation. Neuroepithelial cells were enriched in pathways associated with neurotransmitter release and signal transduction, consistent with their potential role in environmental sensing and intercellular signaling. Among hemocytes, precursor hemocytes showed significant enrichment in “proximal tubular bicarbonate reclamation”, “mitophagy”, and the “IL‐17 signaling pathway”, indicating involvement in ion homeostasis, mitochondrial quality control, and immune regulation. In contrast, semi‐granulocytes were enriched in “insulin secretion”, “nitrogen metabolism”, and the “Toll‐like receptor signaling pathway”, highlighting their coordinated roles in metabolic regulation, ammonia homeostasis, and innate immune defense.

These findings demonstrate that distinct cell populations coordinate the adaptive response to alkalinity stress through integrated regulation of transport processes, metabolic pathways, acid‐base balance, and immune functions.

### 2.3 Validation of Marker Genes and Gene Set Scoring in Gill Cells and Hemocytes

To validate the accuracy of scRNA‐seq–based cell type annotations and their spatial localization within tissues, representative marker genes from key cell clusters were selected for fluorescence in situ hybridization (Figure 3A, B). Probe sequences are provided in Table S4. In gill tissues, *V‐ATPase*, a marker associated with ion transport, was used to label pillar cells. Its signal was predominantly distributed along longitudinally arranged cells within the gill filaments, forming a continuous strip‐like pattern (Figure 3A(a)). *VEGFR1* and *LPHN2* exhibited specific fluorescence signals in a limited number of discrete cells, corresponding to pericytes and endothelial cells, respectively (Figure 3Ab–c). In addition, *proPO*, *LASP1*, and *AChe* displayed distinct and specific expression patterns, marking hemocytes, nephrocytes, and neuroepithelial cells within the gill tissue, respectively (Figure 3A(d)–(f)). These results confirm the spatial specificity of marker gene expression in gill tissues. In hemocytes, *RAB11FIP4* and *proPO* were validated as marker genes for semi‐granulocytes and granulocytes, respectively. *RAB11FIP4* showed strong and specific fluorescence signals in a subset of hemocytes, whereas proPO exhibited robust expression in a distinct population (Figure 3B(a), (b)). These findings further support the reliability of cell type annotation at the tissue level.

**FIGURE 3:**
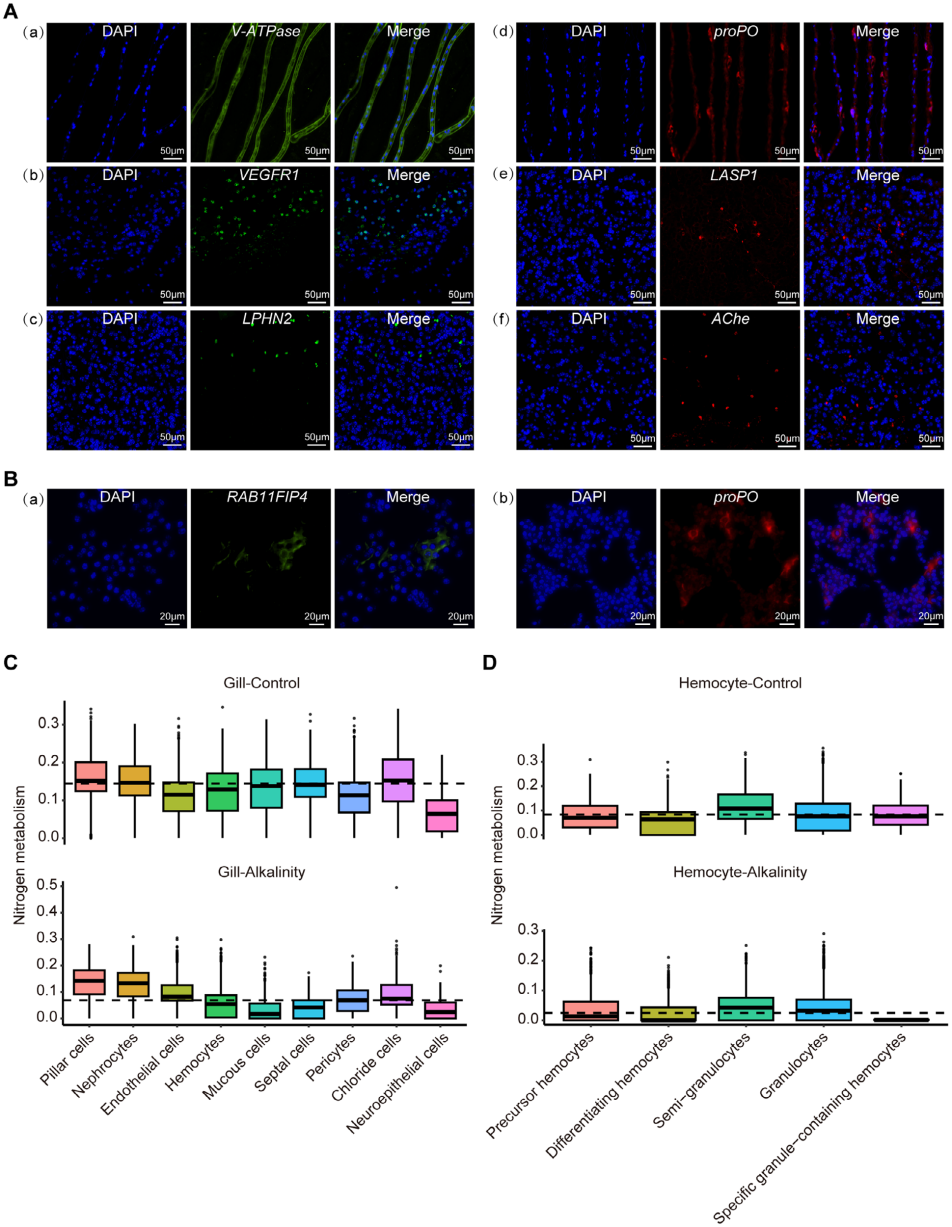
Validation of marker genes and gene set scoring in gill and hemocyte populations. (A) Fluorescence in situ hybridization (FISH) validation of representative marker genes in gill tissue: a) *V‐ATPase* for pillar cells, b) *VEGFR1* for pericytes, c) *LPHN2* for endothelial cells, d) *proPO* for gill‐associated hemocytes, e) *LASP1* for nephrocytes, and f) *AChe* for neuroepithelial cells; nuclei were counterstained with DAPI. (B) FISH validation of representative marker genes in hemocytes: a) *RAB11FIP4* for semi‐granulocytes and b) *proPO* for granulocytes; nuclei were counterstained with DAPI. (C, D) Nitrogen metabolism gene set scores across gill cells and hemocytes. Scale bars: 50 µm in A; 20 µm in B.

Gene set scoring further revealed pronounced cell type‐specific functional differentiation under alkalinity stress. Under stress conditions, pillar cells maintained relatively high activity in nitrogen metabolism and ABC transporter pathways, suggesting key roles in nitrogen metabolic homeostasis and transmembrane transport during alkaline challenge (Figure 3C; Figure S5A). Nephrocytes exhibited the highest enrichment scores in the collecting duct acid secretion pathway, indicating a functional bias toward acid‐base regulation and ion homeostasis (Figure S5A). Among hemocytes, semi‐granulocytes showed not only elevated nitrogen metabolism activity but also strong enrichment in phagosome and Toll‐like receptor signaling pathways (Figure 3D; Figure S5B), suggesting a dual role in metabolic adaptation and innate immune responses under stress conditions. These results demonstrate that alkalinity stress does not induce a uniform cellular response but instead promotes functional specialization among distinct cell populations, particularly pillar cells, nephrocytes, and semi‐granulocytes, in coordinating transport regulation, acid‐base balance, and immune defense.

### 2.4 Pseudotime Analysis of Pillar Cells and Semi‐granulocytes

To characterize the dynamic responses of key cell populations to alkalinity stress, we performed pseudotime trajectory analysis of gill pillar cells and hemocyte semi‐granulocytes (Figure 4A, F). Pillar cells followed a largely unbranched trajectory (Figure 4B), indicating a continuous transcriptional transition rather than divergence into distinct cell fates. Whereas control cells were predominantly located at the early stage of the trajectory (Figure 4C), alkalinity‐stressed cells shifted toward intermediate and late pseudotime states (Figure 4D), suggesting that carbonate alkalinity stress drives pillar cells from a homeostatic state toward an adaptive stress‐response program. Genes associated with pseudotime progression were significantly enriched in the “collecting duct acid secretion”, “oxidative phosphorylation”, “glycolysis/gluconeogenesis”, “carbon metabolism”, and “nitrogen metabolism” pathways (Figure 4E), indicating coordinated enhancement of proton‐coupled ion transport, energy metabolism, and nitrogen metabolic regulation to maintain ionic and intracellular pH homeostasis under alkaline conditions. Concurrent enrichment of the “tight junction”, “focal adhesion”, and “apoptosis” pathways further suggests structural remodeling of the gill epithelium during stress adaptation. Together, these findings identify pillar cells as a central functional population mediating gill adaptation to carbonate alkalinity stress.

**FIGURE 4:**
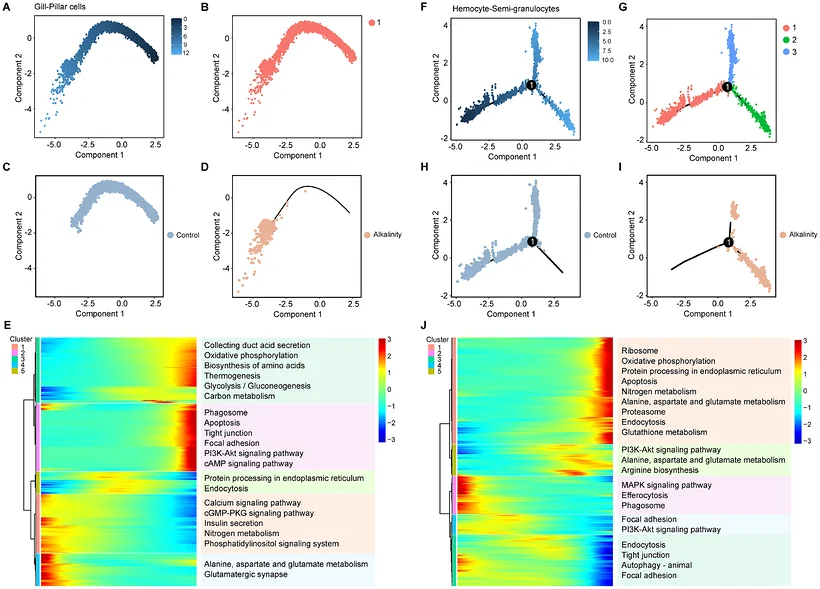
Pseudotime analysis of pillar cells and semi‐granulocytes. (A–D) Pseudotime trajectory analysis of pillar cells in the gills. A) Cells colored by pseudotime. B) Cells colored by trajectory state. C, D) Distribution of cells from the control and alkalinity‐stressed groups, respectively. (E) Heatmap showing pseudotime‐associated gene expression in pillar cells, grouped into five clusters. Representative enriched pathways for each cluster are shown on the right. (F–I) Pseudotime trajectory analysis of semi‐granulocytes in hemocytes. F) Cells colored by pseudotime. G) Cells colored by trajectory state. H, I) Distribution of cells from the control and alkalinity‐stressed groups, respectively. (J) Heatmap showing pseudotime‐associated gene expression in semi‐granulocytes, grouped into five clusters. Representative enriched pathways for each cluster are shown on the right.

In contrast, the pseudotime trajectory of semi‐granulocytes resolved into three distinct cellular states (Figure 4G). Control cells were primarily distributed within States 1 and 2 (Figure 4H), whereas alkalinity‐stressed cells were predominantly enriched in State 3 (Figure 4I), indicating that semi‐granulocytes undergo state‐specific functional reprogramming rather than a single continuous transition during the stress response. Genes associated with pseudotime progression were enriched in “oxidative phosphorylation”, “protein processing in the endoplasmic reticulum”, and the “proteasome” pathway, consistent with enhanced mitochondrial energy metabolism and proteostasis (Figure 4J). Additional enrichment of the “phagosome”, “efferocytosis”, “MAPK signaling”, and “endocytosis” pathways suggests activation of phagocytic clearance and immune signaling. Enrichment of “nitrogen metabolism” together with “alanine, aspartate and glutamate metabolism” further indicates that semi‐granulocytes contribute to the adaptive regulation of hemolymph nitrogen metabolism under alkalinity stress (Figure 4J). These findings demonstrate that coordinated functional remodeling of the gill epithelium and hemocyte populations underpins the integrated physiological response to carbonate alkalinity stress.

### 2.5 Co‐Expression Network Analysis and the Expression and Localization of GDH Under Alkaline Stress

To further investigate gene regulatory responses to alkalinity stress at the single‐cell level, weighted gene co‐expression network analysis (WGCNA) was performed on gill cells and hemocytes. Using a minimum module size of 50 genes, multiple co‐expression modules were identified, and the top 20 modules were selected for subsequent analysis, each containing 129–3,704 genes (Figure 5A, B). Correlation analysis revealed generally low inter‐module correlations, indicating strong modular independence and supporting the robustness of the network construction (Figure S6). Among the identified modules, the salmon and blue modules contained the largest numbers of genes, with 3,704 and 1,751 genes, respectively (Figure 5B). Expression pattern analysis showed that the salmon module was predominantly enriched in hemocytes and exhibited high expression in differentiating hemocytes, whereas the blue module was mainly enriched in gill cells and showed strong expression in pillar cells (Figure S7). Further examination of transcription factor composition revealed that the salmon and blue modules contained 182 and 104 transcription factors, respectively, the highest among all modules, suggesting that these modules play central regulatory roles in the response to alkalinity stress (Figure S8; Table S5).

**FIGURE 5:**
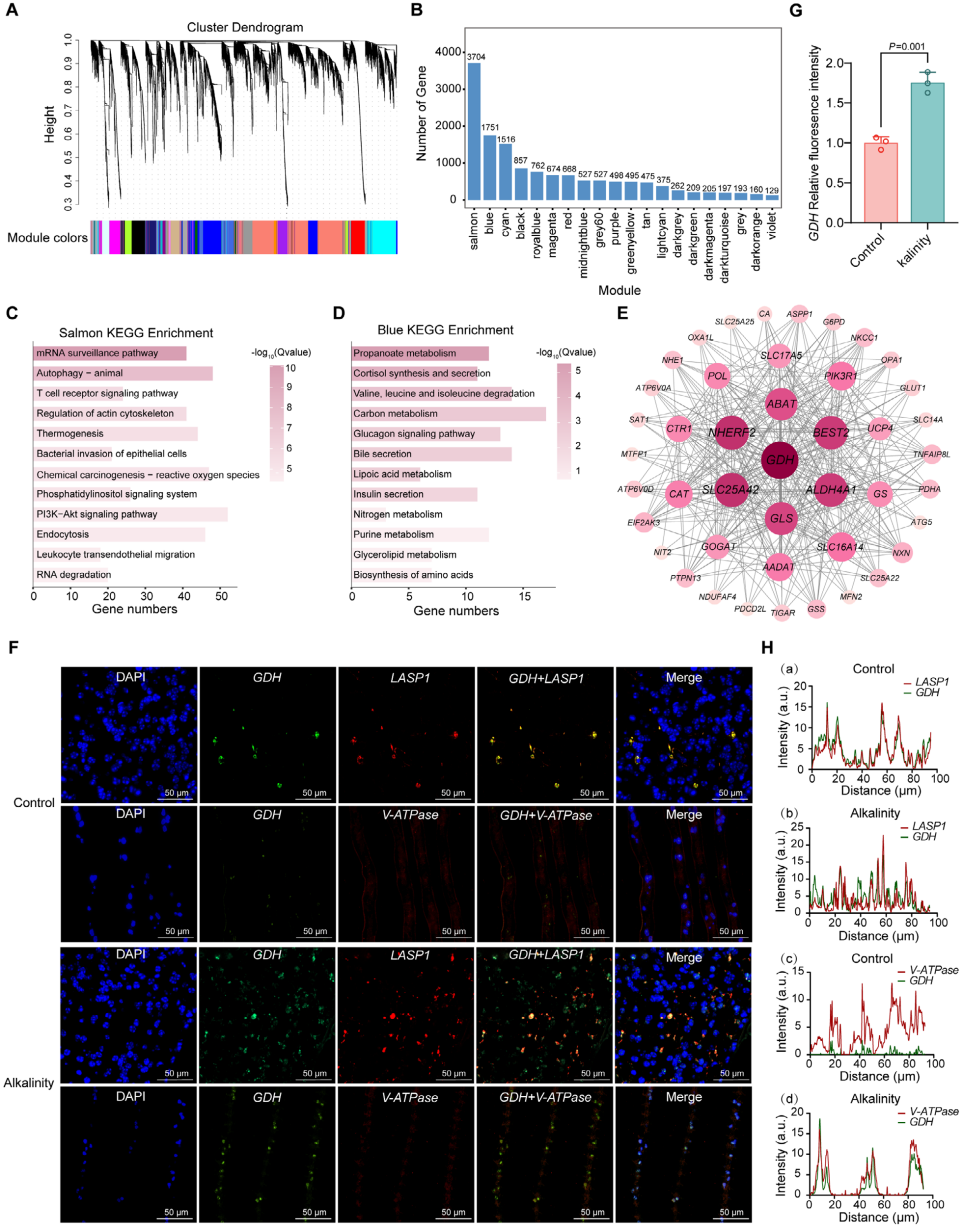
Co‐expression network analysis and GDH cellular localization in the gill. (A) Hierarchical clustering dendrogram of genes and corresponding module assignment in the weighted gene co‐expression network analysis (WGCNA) where different colors represent distinct co‐expression modules. (B) Number of genes contained in the major co‐expression modules. (C, D) KEGG enrichment analysis of genes in the salmon and blue modules. (E) Co‐expression network constructed from hub genes within the key module. (F) Dual fluorescence localization analysis in the gill showing the spatial distribution of GDH with *LASP1* or *V‐ATPase*; nuclei were counterstained with DAPI (blue), GDH signals are shown in green, and *LASP1* or *V‐ATPase* signals are shown in red; merged images indicate co‐localization. (G) Quantification of GDH fluorescence intensity. (H) Line‐scan profiles of *GDH* and *LASP1* or *V‐ATPase* signals. Scale bars: 50 µm in F.

KEGG enrichment analysis further revealed that pathways within the salmon module were predominantly associated with immune responses, cellular repair, and antioxidant processes, indicating a critical role in enabling hemocytes to cope with alkalinity stress (Figure 5C). In contrast, the blue module was mainly enriched in pathways related to metabolic processes, endocrine regulation, and amino acid metabolism, suggesting its involvement in adaptive regulation in gill cells and the maintenance of physiological homeostasis under alkaline conditions (Figure 5D). Based on the identified hub genes within these modules, a co‐expression network was subsequently constructed (Figure 5E). Within this network, *GDH* emerged as a central hub gene and exhibited strong co‐expression relationships with several functionally relevant genes. These included *GLS*, *GOGAT*, and *GS* in nitrogen and amino acid metabolism, *SLC25A42*, *SLC25A22*, and *UCP4* in mitochondrial transport and energy metabolism, and *NHERF2*, *BEST2*, and *NKCC1* in ion and acid‐base regulation. These associations suggest that *GDH* may contribute to the coordinated regulation of nitrogen metabolism, mitochondrial function, and ionic homeostasis during alkalinity stress.

To further characterize *GDH*, the full‐length cDNA was cloned from *M. hainanense*. The open reading frame (ORF) was 1,662 bp in length and encoded a protein of 554 amino acids with a predicted molecular weight of approximately 61 kDa (Figure S9). Phylogenetic analysis showed that *GDH* from *M. hainanense* clustered with homologs from other crustacean species and shared high sequence similarity with those of closely related taxa (Figure S10). To explore the potential upstream regulation of *MhGDH*, the 2‐kb region upstream of the annotated ATG start codon was analyzed. Several putative cis‐regulatory elements and transcription factor‐binding sites were identified, including a predicted USF2‐binding site at ‐1178 to ‐1168 bp corresponding to 5′‐GGCAGGTGATA‐3′ (Figure S11; Table S6). Subsequently, the cellular localization of *MhGDH* was performed by co‐localization analysis using *LASP1* and *V‐ATPase* as markers for nephrocytes and pillar cells, respectively. *GDH* signals were detected in both LASP1‐positive and V‐ATPase‐positive cells. Notably, under alkaline stress, a proportion of *GDH* signals did not fully overlap with either marker, suggesting that *GDH* is also present in additional cell types (Figure 5F, H). Quantitative fluorescence analysis further demonstrated that *GDH* expression was significantly upregulated in the alkalinity stress group compared with the control group (Figure 5G). These results indicate that the *GDH* response to alkalinity stress is not restricted to a single cell population but instead involves coordinated regulation across multiple cell types.

### 2.6 Effects of GDH Interference on Ammonia Assimilation Under Alkalinity Stress

The ammonia assimilation pathway is illustrated in Figure 6A. Analysis of gene expression across eight tissues showed that Rh‐associated glycoprotein (Rhag) was predominantly expressed in the gills, whereas glutamine synthetase (GS) was highly expressed in both the gills and antennal glands. In contrast, *GDH* and glutamate synthase (*GOGAT*) exhibited consistently high transcript levels across all examined tissues (Figure S12), suggesting central roles in ammonia assimilation. To investigate the functional significance of GDH during alkalinity stress, *M. hainanense* glutamate dehydrogenase (*MhGDH*) was silenced by double‐stranded RNA (dsRNA)‐mediated RNA interference. Western blot analysis confirmed efficient knockdown, with GDH protein levels reduced to 36.5% and 22.7% of control levels at 48 and 96 h post‐injection, corresponding to knockdown efficiencies of 63.5% and 77.3%, respectively. In contrast, GDH protein abundance remained unchanged in the dsEGFP group at both time points (Figure 6C, E), confirming the specificity of the RNAi treatment. GDH knockdown markedly reduced the tolerance of *M. hainanense* to alkalinity stress. Mortality progressively increased in the dsGDH‐Alkalinity group during alkalinity exposure, resulting in significantly lower survival than in the dsEGFP‐Alkalinity group (Figure 6B). Histological analysis further revealed severe gill injury following GDH knockdown, characterized by extensive cellular aggregation, disruption of gill architecture, infiltration of hemocytes into intercellular spaces, and increased apoptosis within the gill filaments (Figure 6H, J, K). Consistent with these pathological changes, transmission electron microscopy showed aggravated ultrastructural damage, including extensive vacuolization, mitochondrial cristae disruption, and loss of membrane integrity in the dsGDH‐Alkalinity group relative to the dsEGFP‐Alkalinity group (Figure 6I). To further assess the effects of GDH knockdown on ammonia assimilation, the expression of key pathway components was examined. GS protein abundance increased under alkalinity stress at both 48 and 96 h but was significantly reduced following GDH knockdown at 48 h before recovering to stress‐induced levels by 96 h (Figure 6D, F). In contrast, Rhag expression remained consistently elevated after GDH knockdown at both time points (Figure 6D, G). These findings demonstrate that GDH is required for effective adaptation to alkalinity stress by preserving gill integrity and maintaining ammonia assimilation, and that disruption of GDH compromises both tissue homeostasis and nitrogen metabolic regulation.

**FIGURE 6:**
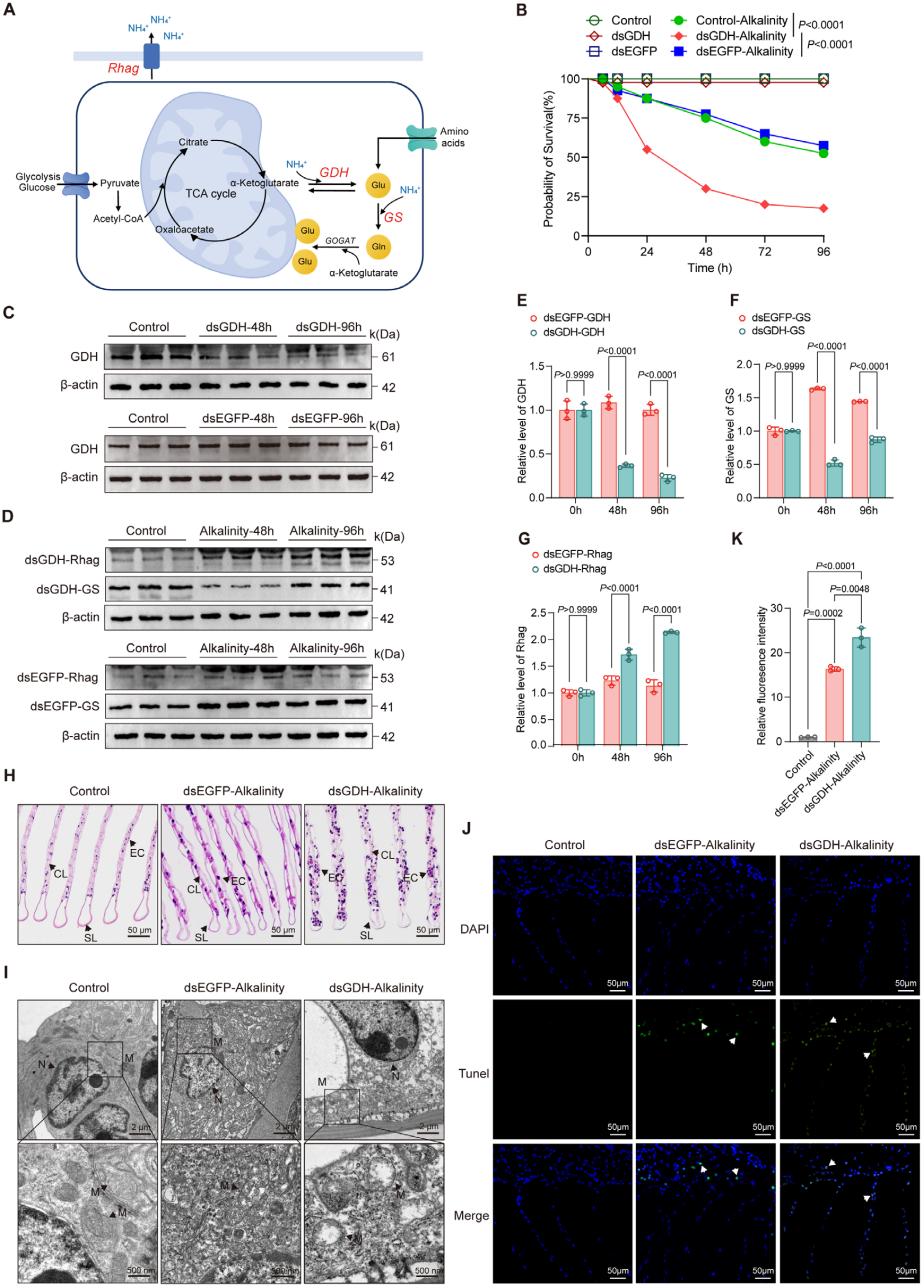
GDH knockdown impairs ammonia assimilation and reduces alkalinity tolerance in *M. hainanense*. (A) Schematic diagram of the proposed ammonia assimilation pathway. (B) Survival curves of the different treatment groups during alkalinity exposure. (C) Western blot analysis of GDH protein abundance at 48 and 96 h after dsGDH or dsEGFP injection. (D) Western blot analysis of GS and Rhag proteins; β‐actin served as the loading control. (E) Quantification of GDH protein levels shown in C. (F, G) Quantification of GS and Rhag protein levels shown in D. (H) HE staining of the gill. (I) TEM images of the gill; boxed regions are shown at higher magnification below. (J) TUNEL staining of the gill where nuclei are stained with DAPI and apoptotic signals are shown in green; arrows indicate TUNEL‐positive cells. (K) Quantification of relative TUNEL fluorescence intensity. Abbreviations: GDH, glutamate dehydrogenase; GS, glutamine synthetase; Rhag, Rh‐associated glycoprotein; GOGAT, glutamate synthase; EC, epithelial cell; CL, capillary lumen; SL, secondary lamella; N, nucleus; M, mitochondrion. Scale bars: 50 µm in H and J; 2 µm in the upper panels of I; 500 nm in the lower panels of I.

### 2.7 Effects of GDH Knockdown on Primary Cell Proliferation, Mitochondrial Function, and Oxidative Stress

To assess the cellular effects of MhGDH knockdown, primary cultures of gill cells and hemocytes were established and subjected to physiological analyses. Morphological observations showed that gill cells in the control group retained intact structure with smooth cell surfaces. In the dsEGFP‐Alkalinity group, cells exhibited mild shrinkage and reduced surface smoothness, whereas these alterations were markedly exacerbated in the dsGDH‐Alkalinity group, where pronounced cell shrinkage and severe morphological disruption were observed (Figure 7A). In contrast, primary hemocytes displayed no obvious morphological differences between the control and dsEGFP‐Alkalinity groups; however, cells in the dsGDH‐Alkalinity group exhibited compromised membrane integrity and localized membrane disruption (Figure 7B).

**FIGURE 7:**
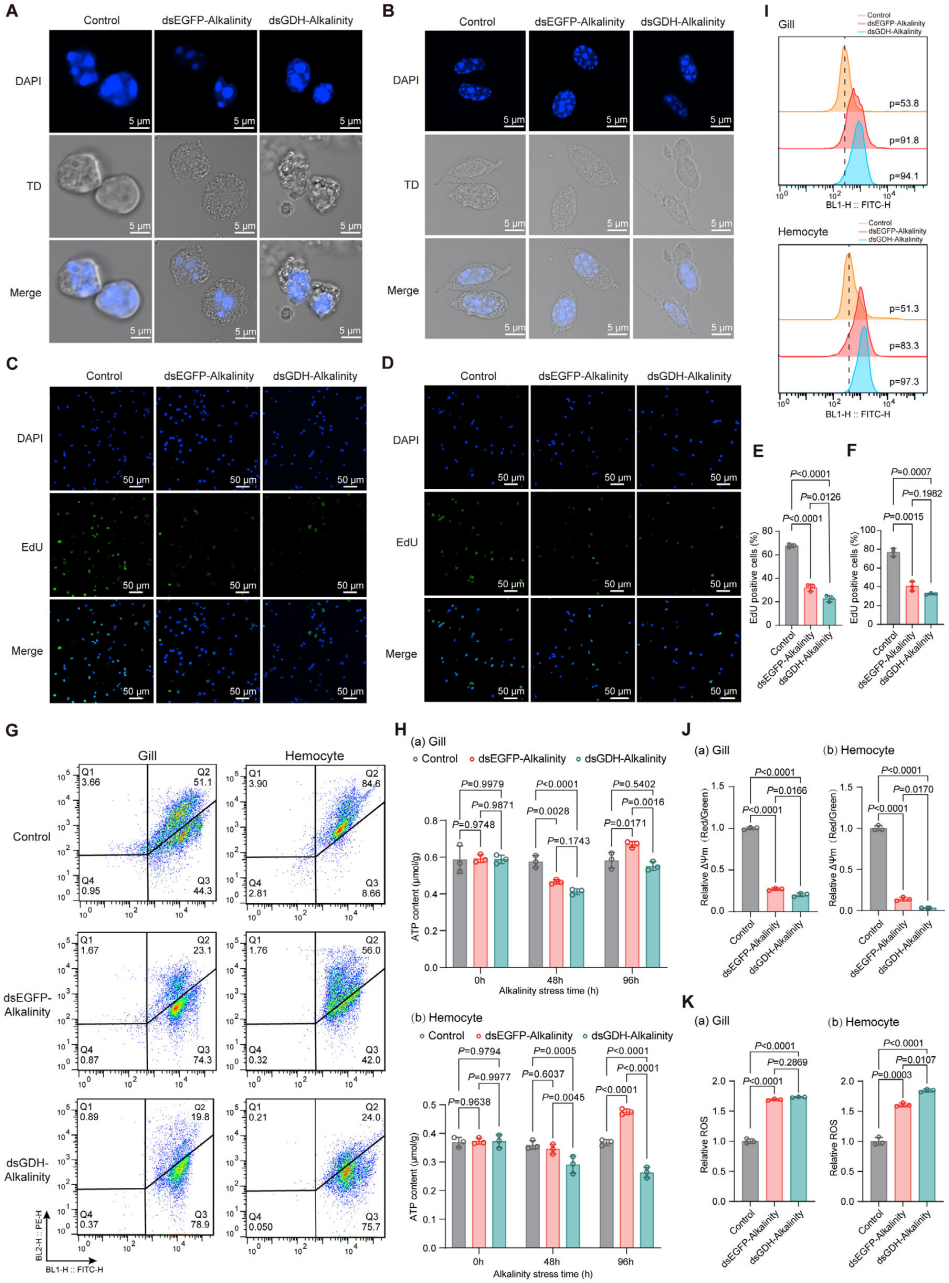
Analyses of primary gill cells and hemocytes after GDH knockdown under alkalinity stress. (A, B) Representative images of primary cultured gill cells and hemocytes where nuclei were stained with DAPI and TD represents transmitted light. (C, D) EdU staining of primary gill cells and hemocytes where nuclei were stained with DAPI and EdU‐positive cells are shown in green. (E, F) Quantification of EdU‐positive cells expressed as a percentage of total DAPI‐stained nuclei in primary gill cells and hemocytes. Panels A, C, and E show primary gill cells, whereas panels B, D, and F show hemocytes. (G) Mitochondrial membrane potential (ΔΨm) in primary gill cells and hemocytes measured by JC‐1 staining. Green fluorescence (BL1‐H) represents JC‐1 monomers, whereas red fluorescence (BL2‐H) represents JC‐1 aggregates. (H) ATP levels in gill tissue and hemocytes at different time points. (I) Representative flow cytometry plots of intracellular reactive oxygen species (ROS) levels in primary cells. (J) Quantification of relative ΔΨm in gill cells and hemocytes. (K) Quantification of relative ROS levels in gill cells and hemocytes. Scale bars: 5 µm in A and B; 50 µm in C and D.

The EdU incorporation assays showed that alkalinity stress suppressed cell proliferation in both gill cells and hemocytes. In gill cells, GDH knockdown further decreased the proportion of EdU‐positive cells relative to the dsEGFP‐Alkalinity group. In contrast, although both alkalinity‐stressed groups exhibited significantly lower EdU‐positive rates than the control group, no significant difference was observed between the dsEGFP‐Alkalinity and dsGDH‐Alkalinity groups in hemocytes (Figure 7C–F). JC‐1 flow cytometric analysis revealed a decrease in mitochondrial membrane potential (ΔΨm) in both stress groups, with a greater reduction in the dsGDH‐Alkalinity group, indicating exacerbated mitochondrial dysfunction (Figure 7G, J). Consistently, ROS levels were elevated under alkalinity stress and further increased upon GDH knockdown, reflecting enhanced oxidative stress (Figure 7I, K). ATP measurements revealed distinct temporal responses in gill cells and hemocytes. In the dsEGFP‐Alkalinity group, ATP levels in both tissues declined initially and partially recovered during prolonged stress. In contrast, GDH knockdown significantly impaired ATP maintenance. In gill cells, ATP levels were significantly lower than those in the dsEGFP‐Alkalinity group at 48 h and showed only partial recovery at 96 h. In hemocytes, ATP levels declined progressively throughout the experimental period and remained consistently lower than those in the dsEGFP‐Alkalinity group (Figure 7H). These findings demonstrate that GDH knockdown exacerbates structural damage in both gill cells and hemocytes, impairs ATP homeostasis, and aggravates mitochondrial dysfunction and oxidative stress during alkalinity stress.

### 2.8 Metabolomics‐Based Analysis of Metabolic Responses and Key Metabolites

Untargeted metabolomic profiling was conducted to characterize metabolic alterations in gill tissues following GDH knockdown. Principal component analysis (PCA) revealed clear separation among the control, dsEGFP‐Alkalinity, and dsGDH‐Alkalinity groups, indicating that alkalinity stress induces substantial metabolic reprogramming, while GDH interference further reshapes the overall metabolic trajectory of the stress response (Figure 8A). Volcano plot analysis showed that, compared with the control group, 198 DEMs were identified in the dsEGFP‐Alkalinity group, including 139 upregulated and 59 downregulated metabolites (Figure 8B(a)). In the dsGDH‐Alkalinity group, 261 DEMs were detected, comprising 194 upregulated and 67 downregulated metabolites (Figure 8B(b)). Direct comparison between the dsGDH‐Alkalinity and dsEGFP‐Alkalinity groups revealed even greater metabolic divergence, with 352 DEMs identified, including 217 upregulated and 135 downregulated metabolites (Figure 8B(c)). These DEMs were subsequently annotated using the KEGG database.

**FIGURE 8:**
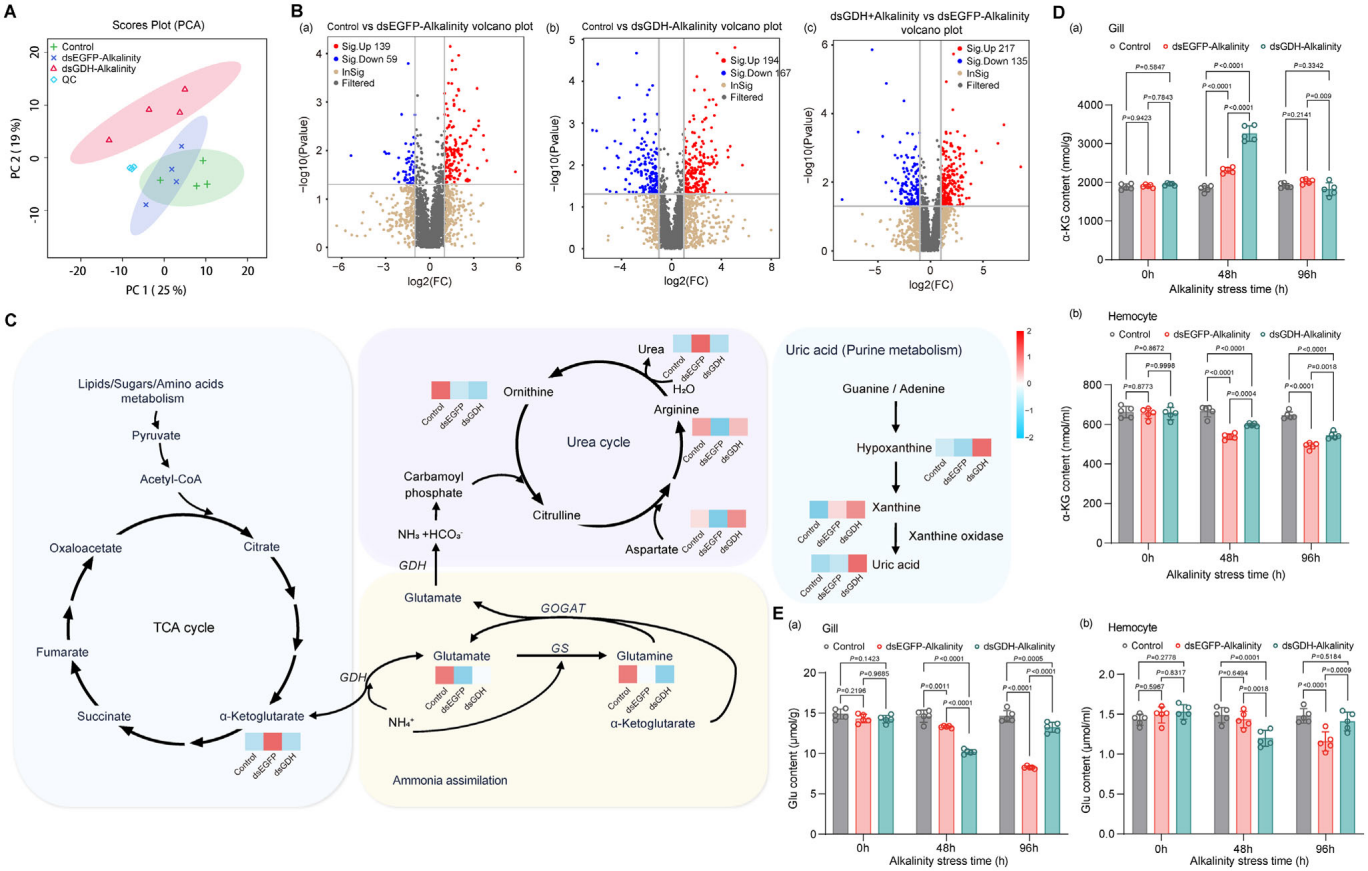
Metabolomic analyses and changes in key metabolites after GDH knockdown under alkalinity stress. (A) PCA score plot of metabolomic profiles. (B) Volcano plots of differential metabolites. (C) Schematic summary of the major metabolic pathways altered under alkalinity stress, including the tricarboxylic acid (TCA) cycle, urea cycle, ammonia assimilation pathway, and purine metabolism; heatmaps indicate relative changes in representative metabolites among the three groups. Both dsEGFP and dsGDH groups were exposed to alkalinity stress, with dsEGFP serving as the dsRNA control and dsGDH representing the GDH‐knockdown group. (D) Quantification of α‐ketoglutarate (α‐KG) levels in the gill and hemocytes at different time points. (E) Quantification of glutamate (Glu) levels in gill tissue and hemocytes at different time points.

The metabolomic analysis revealed extensive reprogramming of pathways associated with energy metabolism and ammonia nitrogen metabolism (Figure 8C). In gill tissue, the key tricarboxylic acid (TCA) cycle intermediate α‐ketoglutarate (α‐KG) was significantly elevated in the dsEGFP‐Alkalinity group compared with the control group at 48 h, with an even greater increase observed in the dsGDH‐Alkalinity group. At 96 h, α‐KG levels in the dsGDH‐Alkalinity group declined relative to those at 48 h (Figure 8D(a)). Correspondingly, glutamate levels in the gills were significantly reduced in the dsGDH‐Alkalinity group at 48 h. At 96 h, the most pronounced decrease in glutamate occurred in the dsEGFP‐Alkalinity group, whereas glutamate levels in the dsGDH‐Alkalinity group remained significantly higher than those in the dsEGFP‐Alkalinity group (Figure 8E(a)). In addition, glutamine levels were markedly reduced in the dsGDH‐Alkalinity group, suggesting adaptive modulation of nitrogen assimilation pathways, such as the GS/GOGAT pathway (Figure 8C). In the dsEGFP‐Alkalinity group, urea levels increased, while ornithine, arginine, and aspartate decreased after 96 h, indicating activation of the urea cycle to mitigate ammonia accumulation. Notably, purine metabolism was enhanced in the dsGDH‐Alkalinity group, as evidenced by increased levels of hypoxanthine, xanthine, and uric acid, suggesting activation of nucleotide catabolism and uric acid production (Figure 8C). In hemocytes, α‐KG levels showed a gradual decline over time in the dsEGFP‐Alkalinity group, whereas relatively elevated levels were maintained in the dsGDH‐Alkalinity group at both 48 and 96 h (Figure 8D(b)). Glutamate levels exhibited less pronounced fluctuations overall, although a more substantial decrease was observed in the dsEGFP‐Alkalinity group at 96 h (Figure 8E(b)).

KEGG enrichment analysis further showed that, compared with the control group, the dsEGFP‐Alkalinity group was significantly enriched in pathways related to lipid, amino acid, and carbohydrate metabolism. In contrast, the dsGDH‐Alkalinity group, when compared with the dsEGFP‐Alkalinity group, exhibited significant enrichment in pathways associated with lipid metabolism, alternative amino acid metabolism, and neuroendocrine signaling regulation (Figure S13), indicating that GDH knockdown further disrupts membrane lipid homeostasis, redox balance, and neural regulatory networks. These findings suggest that, under alkalinity stress, the organism adapts by modulating energy metabolism and enhancing ammonia nitrogen metabolism. In contrast, MhGDH knockdown promotes purine degradation, reduces ammonia assimilation capacity, and ultimately exacerbates metabolic disturbances.

## 3 Discussion

### 3.1 Alkalinity Stress Induces an Imbalance in Ammonia Nitrogen Metabolism in *M. hainanense*

To elucidate the impact of carbonate alkalinity stress on ammonia metabolism in *M. hainanense*, it is first necessary to determine whether ammonia excretion is impaired. In crustaceans, the majority of ammonia is excreted across the gill epithelium, whereas the contribution of the antennal gland via urinary excretion is minimal [32]. For example, in the blue crab *Callinectes sapidus*, urinary ammonia accounts for less than 2% of total ammonia excretion, with the remaining nitrogenous waste either excreted as urea or uric acid or retained for internal metabolic processes [33]. These findings highlight the central role of the gills in maintaining ammonia homeostasis in crustaceans. Under high alkalinity conditions, the conversion of NH_3_ to NH_4_^+^ and its subsequent excretion are inhibited, leading to disruption of hemolymph acid‐base balance and structural damage to the gills. These changes impair ion transport and reduce ammonia excretion efficiency, ultimately resulting in the accumulation of ammonia in the hemolymph [24, 29]. Consistent with this, more severe structural damage and pronounced mitochondrial abnormalities are observed in gill tissues compared with antennal glands, likely due to the direct exposure of gills to the external environment. Hemolymph NH_4_^+^ levels increase progressively with prolonged alkalinity stress, a pattern also reported in *L. vannamei* [24]. All prawns were fasted throughout the experiment to minimize variation in dietary nitrogen input, as feeding can rapidly alter hemolymph ammonia levels and ammonia excretion in crustaceans [34]. Meanwhile, ammonia concentrations in the culture water remained stable from 6 to 96 h, suggesting that the progressive increase in hemolymph NH_4_^+^ was unlikely to be driven by changes in waterborne ammonia. Moreover, NH_4_^+^ accumulation predominantly occurs in gill tissues, and higher expression levels of Rhag in the gills relative to the antennal glands further support their primary role in ammonia excretion [13, 32]. Notably, alkalinity stress also induces alterations in urea metabolism. Together, these findings suggest that when gill‐mediated ammonia excretion is compromised, enhanced urea metabolism may act as a compensatory mechanism to mitigate ammonia toxicity under alkaline conditions. Similarly, in fishes exposed or adapted to saline–alkaline waters, impaired ammonia excretion is often accompanied by compensatory nitrogen detoxification responses, including enhanced glutamine synthesis and modulation of urea‐related metabolism [35, 36]. Together with our findings, these observations suggest that aquatic animals may share conserved adaptive strategies to cope with alkalinity‐induced impairment of ammonia excretion by reprogramming nitrogen metabolic pathways to reduce internal ammonia toxicity.

### 3.2 ScRNA‐Seq Reveals Heterogeneous Responses of Gill and Hemocyte Cells to Alkalinity Stress

Previous studies of alkalinity stress in crustaceans have largely relied on physiological, biochemical, or bulk omics approaches at the tissue level, leaving a gap in understanding cell‐type‐specific functional differentiation and intercellular interactions under stress conditions [37, 38, 39]. To address this limitation, a single‐cell atlas of gill and hemocyte populations in *M. hainanense* under acute alkalinity stress was constructed, providing high‐resolution insights into ammonia metabolism and immune responses. Alkalinity stress led to a marked reduction in the proportion of pillar cells in the gills, accompanied by a substantial increase in hemocytes and endothelial cells. During single‐cell preparation, we observed that cells isolated from alkalinity‐stressed gills were more fragile and exhibited a more rapid loss of viability during tissue dissociation, suggesting that dissociation‐based scRNA‐seq may underestimate highly damaged cell populations in severely injured gill tissues [40]. Nevertheless, the increased abundance of hemocytes and endothelial cells, together with the pronounced transcriptional and functional remodeling of pillar cells, indicates substantial reorganization of the gill cellular landscape in response to alkalinity stress. These findings further support the notion that pillar cells are a primary target of alkalinity stress and undergo marked functional reprogramming to maintain gill homeostasis under alkaline conditions. This observation is consistent with findings in *E. carinicauda*, where pillar cells were identified as highly sensitive to environmental stress, showing significant perturbations in ion regulation, acid‐base balance, and ammonia excretion, together with disrupted energy metabolism [29, 41]. The increased abundance of hemocytes and endothelial cells may reflect local immune activation following damage to the gill epithelial barrier, promoting hemocyte recruitment and indicating a coordinated role in maintaining tissue integrity and the local immune microenvironment [42, 43, 44]. Similar phenomena of gill injury accompanied by hemocyte infiltration have been reported in other crustacean species [45]. Functional annotation further revealed distinct roles of different cell types under alkalinity stress. Pillar cells were primarily associated with energy metabolism and transmembrane transport, supporting compensatory adjustments in ion and substrate exchange, potentially modulated by neurotransmitter signaling. In contrast, nephrocytes and septal cells showed enhanced activity in pathways related to acid‐base homeostasis, metabolic reprogramming, and phagocytic processes, highlighting their contributions to maintaining cellular homeostasis and defense. These findings demonstrate that gill adaptation to alkalinity stress is mediated by coordinated, cell‐type‐specific functional specialization.

At the hemocyte level, acute alkalinity stress resulted in a marked increase in the proportions of precursor and differentiating hemocytes, accompanied by a reduction in semi‐granulocytes, indicating activation of hematopoiesis and remodeling of hemocyte lineages. Previous studies have shown that circulating hemocyte numbers decline rapidly following stress or infection, and their recovery depends not only on hematopoietic replenishment from the HPT but also on the rapid mobilization of sessile hemocytes, thereby maintaining overall hemocyte homeostasis [46, 47, 48]. Meanwhile, single‐cell analyses in shrimp have delineated hemocyte differentiation trajectories and identified TGase as a key marker of immature or early‐stage hemocytes [26, 48, 49]. Consistent with these findings, an increased proportion of *HMGA1*‐positive precursor hemocytes was observed; *HMGA1* is typically associated with highly proliferative cell populations, such as embryonic cells [50, 51]. This was accompanied by a concurrent expansion of differentiating hemocytes marked by TGase and histone‐related genes, further supporting the notion that alkalinity stress promotes hemocyte proliferation and differentiation. Notably, hemocyte responses were not limited to immune activation, such as phagocytosis and inflammatory signaling, but also involved enhanced PPAR signaling, mitophagy, and broader metabolic reprogramming. These results suggest that immune effector functions and metabolic adaptation are tightly coordinated in hemocytes during the response to alkalinity stress.

Previous studies have demonstrated functional interactions between gills and hemocytes in shrimps, likely facilitated by the open circulatory system of crustaceans, which enables circulating hemocytes to access and interact with gill tissues [52, 53]. A similar activation of immune‐ and phagosome‐related pathways has been reported in *E. carinicauda* under alkalinity stress [54]. In fish, immune responses to alkalinity stress involve both barrier tissues, such as the gills, and systemic immune organs, such as the spleen [55, 56]. In contrast, crustaceans lack a single specialized immune organ, and their immune defense is mediated primarily by circulating hemocytes, with the gills also contributing to local immune responses [54, 57]. Consistent with this concept, our comparative analyses indicate that alkalinity stress elicits coordinated responses in both gill cells and circulating hemocytes. The predicted cell–cell communication network further identifies pericytes and endothelial cells as potential communication hubs linking gill‐resident cells with hemocytes. Molecular docking of RGMb–NEO1 and IL1RAPL1–PTPRF provided additional structural support for these predicted interactions, although further experimental confirmation is needed. Together with the enrichment of immune‐related signaling pathways in these cell populations, these findings suggest that pericytes and endothelial cells contribute to coordinating local immune responses during alkalinity stress. In addition, enrichment of the phagosome pathway in nephrocytes and septal cells indicates that these gill cell populations may participate in defense‐related processes. In the hemocyte compartment, the expansion of precursor and differentiating hemocytes, together with the enrichment of immune‐ and metabolism‐related pathways in semi‐granulocytes, supports a coordinated role for circulating hemocytes in both immune defense and metabolic adaptation [57]. These findings suggest that gill and hemocyte responses are functionally integrated during alkalinity stress, with pericytes and endothelial cells potentially serving as key mediators of intercellular communication between the gill and the circulating immune system.

Based on WGCNA, the adaptive response of gill cells to alkalinity stress appears to be tightly linked to metabolic reprogramming and the regulation of nitrogen metabolism. Among the identified modules, *GDH* emerged as a central hub gene and is likely to play a key role in maintaining cellular homeostasis. Its differential expression across multiple cell clusters, together with the enrichment of nitrogen metabolism pathways, further supports its role as a critical mediator of the adaptive response to alkalinity stress. GDH is a mitochondrial enzyme that catalyzes the reversible conversion between L‐glutamate and α‐ketoglutarate, thereby coupling amino acid catabolism with ammonia assimilation. This positions GDH at the interface of nitrogen and energy metabolism, where it contributes to ammonia detoxification and the prevention of hyperammonemia [58, 59]. As a core component of the nitrogen metabolic network, GDH has been shown to maintain physiological homeostasis in crustaceans under environmental challenges such as salinity, ammonia stress, and alkalinity by regulating amino acid metabolism, ammonia detoxification, and osmoregulatory processes [24, 60, 61]. In this study, promoter analysis identified a putative USF2‐binding site in the upstream region of *MhGDH*. USF2 is an E‐box‐binding transcription factor associated with mitochondrial function and energy homeostasis, suggesting that it may contribute to stress‐induced *MhGDH* expression [62, 63]. However, direct binding and transcriptional regulation require further experimental validation. The cellular localization of *GDH* was markedly altered under alkalinity stress, shifting from predominant expression in nephrocytes under control conditions to co‐expression in both nephrocytes and pillar cells. This redistribution coincided with a pseudotime shift in pillar cells, with control cells predominantly occupying the early trajectory and alkalinity‐stressed cells progressing toward later pseudotime states. These transcriptional states were characterized by enrichment of pathways associated with energy metabolism, nitrogen metabolism, and stress‐ and immune‐related responses, indicating extensive functional reprogramming of pillar cells during alkalinity stress. Similar changes in the TCA cycle and in carbohydrate and lipid metabolism have been reported in *E. sinensis* and *L. vannamei* under carbonate alkalinity stress [64, 65]. Mitochondrial and lipid metabolic adjustments have also been observed in *Gymnocypris przewalskii* under saline–alkaline conditions, suggesting that these species share similar energetic strategies to sustain ion regulation and cellular homeostasis [35]. Accordingly, the induction of *GDH* expression in pillar cells may represent a cell type‐specific adaptive mechanism that coordinates ammonia assimilation with proton‐coupled ion transport and mitochondrial energy metabolism, thereby supporting cellular homeostasis under alkaline conditions. In addition, the detection of *GDH* signals in other cell populations indicates that its function is not restricted to a single cell type but instead involves coordinated activity across multiple cell types, contributing to a systemic adaptive response to alkalinity stress.

### 3.3 Role of GDH in Metabolic Regulation and Adaptive Responses of *M. hainanense* to Alkalinity Stress

To further elucidate the role of GDH in the response to alkalinity stress, *MhGDH* was silenced, revealing a significantly higher mortality rate in the dsGDH‐Alkalinity group compared with the dsEGFP‐Alkalinity group, indicating that GDH deficiency markedly reduces tolerance to alkaline conditions. Histological analysis showed pronounced epithelial hyperplasia and clustered aggregation in gill filaments and lamellae following GDH knockdown. This response is generally interpreted as a compensatory remodeling process under carbonate alkalinity stress, involving expansion of the ion‐transporting surface to facilitate processes such as Cl^−^/HCO_3_^−^ exchange and maintain ionic and acid‐base homeostasis [66, 67, 68]. Consistent with these tissue‐level observations, primary gill cells and hemocytes from *GDH*‐deficient individuals exhibited more severe shrinkage under stress, with hemocytes also showing pronounced membrane disruption, suggesting impaired osmoregulatory capacity and reduced membrane stability. These structural and functional impairments are likely associated with mitochondrial dysfunction and enhanced oxidative stress.

Mitochondria serve not only as the central sites of ATP production but also as key regulators of ROS homeostasis. Disruption of ΔΨm and electron transport chain function reduces oxidative phosphorylation efficiency and promotes excessive ROS accumulation, thereby amplifying oxidative damage [69, 70]. Consistent with these observations, GDH knockdown further decreased ΔΨm and increased ROS production in both gill cells and hemocytes, indicating that loss of GDH function compromises mitochondrial integrity and antioxidant capacity, thereby exacerbating cellular damage under alkalinity stress. Because cellular energy metabolism is closely coupled to cytoskeletal organization, ATP depletion can impair actin remodeling and compromise membrane‐associated structures and barrier integrity [71]. Alkalinity stress imposes substantial energetic demands on both gill cells and hemocytes as they maintain ion transport and cellular homeostasis. Consistent with this, the enrichment of oxidative phosphorylation, glycolysis/gluconeogenesis, and carbon metabolism pathways in pillar cells highlights extensive metabolic reprogramming to meet these increased energy demands. The transient decline followed by partial recovery of ATP levels in the dsEGFP‐Alkalinity group likely reflects an initial disruption of energy balance followed by metabolic compensation, consistent with previous reports of energy metabolic disturbances in crustacean gills under carbonate alkalinity stress [64, 65]. The effects of GDH knockdown on ATP homeostasis differed between gills and hemocytes. Gill ATP levels showed partial recovery at 96 h, whereas ATP levels in hemocytes continued to decline, suggesting that gill cells retain a limited capacity for metabolic compensation, while hemocytes are more vulnerable to sustained energy depletion. These findings indicate that GDH plays a critical role in preserving mitochondrial function and ATP homeostasis during adaptation to alkalinity stress.

In addition to its role in antioxidant defense, GDH may also critically influence ammonia nitrogen metabolism under alkalinity stress. Under elevated alkalinity or ammonia conditions, crustaceans mitigate internal ammonia accumulation by enhancing excretion and activating detoxification pathways such as urea synthesis [72, 73]. Consistent with this, the dsEGFP‐Alkalinity group showed a marked increase in urea content, suggesting a partial diversion of nitrogen flux toward urea production to reduce ammonia toxicity. In contrast, urea levels were reduced in the dsGDH‐Alkalinity group, accompanied by the accumulation of L‐aspartate, L‐ornithine, and arginine, indicating that GDH knockdown may impair urea cycle function. Moreover, the more pronounced elevation of uric acid in the dsGDH‐Alkalinity group suggests a compensatory shift toward purine degradation to eliminate excess nitrogen. Enrichment of glycerophospholipid metabolism following GDH interference further indicates disturbances in membrane lipid metabolism, which may compromise membrane stability and the adaptive capacity of membrane‐associated structures [74]. WGCNA network analysis showed that *GDH* was closely co‐expressed with *GS* and *GOGAT*, suggesting a potential link between the GDH and GS/GOGAT pathways. GDH catalyzes the conversion of α‐KG and NH_4_^+^ to glutamate, whereas GS incorporates NH_4_^+^ into glutamine using glutamate, and GOGAT regenerates glutamate from glutamine and α‐KG, thereby maintaining the glutamate pool required to sustain ammonia assimilation [60, 75]. Therefore, GDH knockdown is likely to compromise adaptation to alkalinity stress by impairing ammonia assimilation and disrupting metabolic homeostasis. Time‐course metabolomic analysis further provided insight into the dynamic metabolic compensation triggered by GDH knockdown. At 48 h post‐interference, suppression of GDH was accompanied by α‐KG accumulation and reduced glutamate levels, suggesting impaired conversion of α‐KG to glutamate following inhibition of GDH activity. This metabolic profile indicates that GDH normally coordinates carbon metabolism with ammonia assimilation to maintain metabolic homeostasis under alkalinity stress. By 96 h, glutamate levels had partially recovered, whereas α‐KG levels declined, suggesting activation of alternative nitrogen assimilation pathways to compensate for the loss of GDH function. Consistent with this interpretation, GS protein abundance was significantly reduced at 48 h after GDH knockdown but recovered by 96 h, supporting the notion that GS‐mediated ammonia assimilation is activated during the later stages of metabolic compensation [24, 60]. In parallel, Rhag expression was also upregulated in the dsGDH‐Alkalinity group, indicating enhanced transmembrane ammonia transport and excretion in gill tissues. Previous studies have demonstrated that Rh proteins are key mediators of branchial ammonia transport, whereas GS‐dependent glutamine synthesis and urea production pathways jointly contribute to ammonia detoxification under conditions of elevated ammonia or alkalinity [13, 60, 76]. These findings suggest that GDH knockdown triggers a compensatory response involving increased GS‐mediated ammonia assimilation and Rhag‐associated ammonia excretion, which may partially compensate for impaired ammonia metabolism resulting from GDH deficiency.

## 4 Conclusions

In summary, this study systematically characterizes the response of *M. hainanense* to acute carbonate alkalinity stress (Figure 9). Elevated alkalinity disrupts ammonia metabolism, leading to impaired excretion and subsequent accumulation of ammonia in the hemolymph, accompanied by gill injury and mitochondrial structural damage. Concurrently, the cellular composition of both gill tissues and hemocytes is markedly altered, with distinct cell types exhibiting specialized responses to alkalinity stress. In particular, pillar cells, nephrocytes, and semi‐granulocytes display functional roles in ion transport, nitrogen metabolism, acid‐base homeostasis, and immune regulation. Further analyses revealed a dynamic redistribution of GDH among gill cell populations under alkalinity stress, indicating functional reprogramming during alkalinity adaptation. Loss of GDH function exacerbated tissue injury and mitochondrial dysfunction, disrupted ammonia metabolism and ATP homeostasis, and ultimately contributed to the increased mortality and reduced alkalinity tolerance of *M. hainanense*. These findings elucidate cell‐type‐specific adaptations to alkalinity stress and highlight the central role of GDH in regulating nitrogen metabolism, providing new mechanistic insights into how aquatic organisms respond to and adapt to challenging environmental conditions.

**FIGURE 9:**
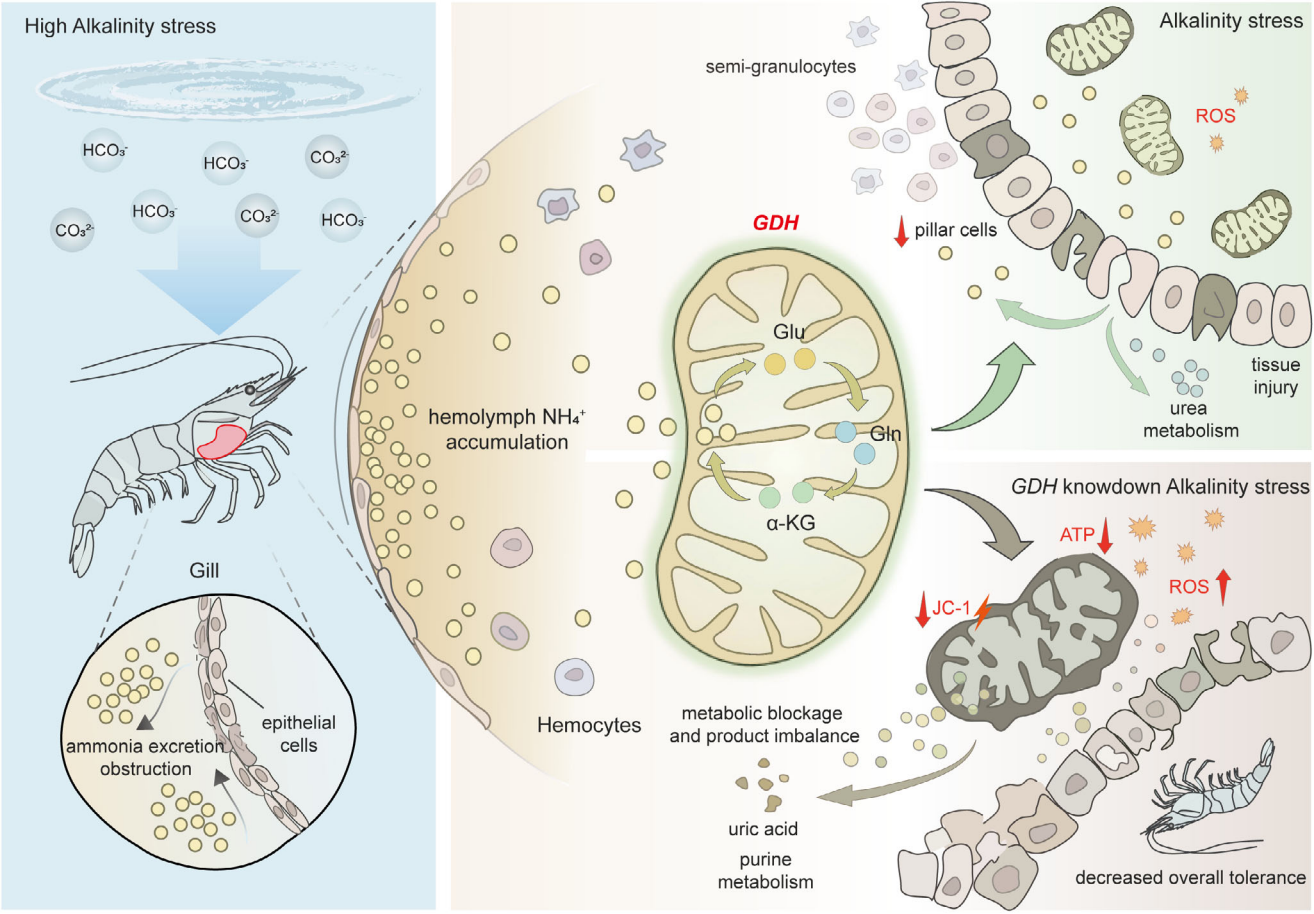
Proposed model of cellular and metabolic responses to alkalinity stress in *M. hainanense*.

## 5 Experimental Section

### 5.1 Experimental Animals

Healthy adult shrimp (*M. hainanense*; approximately 10 g) were collected from the Nandu River, Hainan Province, China. Prior to experimentation, animals were acclimated for one week in aerated tanks maintained at 25°C and fed daily with commercial pellet feed. All experimental procedures were conducted in accordance with the guidelines of the Committee on Laboratory Animal Care and Use of Shanghai Ocean University (Shanghai, China) and were approved under protocol number NJ202306002.

### 5.2 Alkalinity Stress Experiment

Aerated tap water (24 h) was used as the control condition. For the experimental groups, alkalinity was adjusted to 10 mmol/L by the addition of NaHCO_3_, and alkalinity levels were measured using a commercial assay kit (Guangdong Huankai Microbial Technology Co., Ltd., Guangdong, China) according to the manufacturer's instructions. To minimize variation in exogenous nitrogen intake, shrimp were fasted for 24 h before exposure and throughout both the 96 h alkalinity stress experiment and the RNAi experiment conducted under alkalinity stress. Water samples were collected at 0, 6, 12, 24, 48, and 96 h. The CK group contained the same experimental water without prawns and served as an animal‐free water control. Shrimp samples were collected from each group at designated time points following stress exposure. Prior to sampling, individuals were anesthetized in an ice–water bath, after which gill tissues and hemolymph were rapidly harvested. Each sample consisted of pooled tissues from three individuals. All collected samples were immediately snap‐frozen in liquid nitrogen and stored at −80°C until subsequent analyses.

### 5.3 Sample Preparation for HE Staining, TUNEL, and TEM

For histological analysis, freshly collected tissues were fixed in 4% paraformaldehyde at 4°C for 24 h, followed by dehydration through a graded ethanol series, clearing in xylene, and paraffin embedding. Sections (5 µm) were prepared and stained using a Hematoxylin and Eosin Staining Kit (Cat. No. 60524ES60; Yeasen Biotechnology, Shanghai, China), after which tissue morphology was examined under a light microscope. For TUNEL analysis, apoptotic cells were detected using a fluorescence‐based kit (YSFluor 640; Yeasen Biotechnology) according to the manufacturer's instructions. For TEM, samples were fixed in 2.5% glutaraldehyde at 4°C for 24 h, rinsed three times with 0.1 M phosphate buffer (pH 7.4), and post‐fixed in 1% osmium tetroxide for 2 h in the dark. Samples were then dehydrated through a graded ethanol series, embedded in Epon 812 epoxy resin, and sectioned into ultrathin slices (≈70 nm). Sections were stained with uranyl acetate and lead citrate and examined using a JEM‐1200EX transmission electron microscope (JEOL Ltd., Tokyo, Japan).

### 5.4 Detection of Ammonium Ion, Urea, ATP, α‐Ketoglutarate, and Glutamate Content

Ammonium ion, glutamate, α‐ketoglutarate, and urea levels were quantified using commercial assay kits according to the manufacturers’ protocols. Ammonium ion and glutamate concentrations were measured using kits from Nanjing Jiancheng Bioengineering Institute (Cat. Nos. A086‐1‐1 and A074‐1‐1; Nanjing, China). ATP content was measured using an ATP Assay Kit (Cat. No. BC0300; Beijing Solarbio Science & Technology Co., Ltd., Beijing, China). α‐Ketoglutarate and urea levels were determined using assay kits from Beijing KeDeng Biotechnology (Cat. Nos. SK111‐1 and SN134‐1; Beijing, China), with urea quantified based on the diacetyl monoxime method.

### 5.5 Semi‐Quantitative RT‐PCR

Total RNA was extracted from tissue samples using TRIzol reagent (Invitrogen, Carlsbad, CA, USA) according to the manufacturer's instructions. For cDNA synthesis, 5 µg of purified RNA was reverse transcribed using Hifair III 1st Strand cDNA Synthesis SuperMix for qPCR (gDNA digester plus; Cat. No. 11141ES10; Yeasen Biotechnology). Gene‐specific PCR amplification was subsequently performed using primers targeting *β‐actin*, *GDH*, *GS*, *GOGAT*, and *Rhag* (Table S3). Amplification products were separated by 1% agarose gel electrophoresis, visualized using a gel documentation system, and quantified with ImageJ software (National Institutes of Health, Bethesda, MD, USA).

### 5.6 Preparation of Gill and Hemocyte Single‐Cell Suspensions

To construct the single‐cell transcriptomic atlas of gill and hemolymph under alkalinity stress, six individuals from both control and experimental groups were sampled at 96 h post‐exposure. Fresh gill tissues and hemolymph were pooled from three individuals per replicate to generate two biological replicates for scRNA‐seq (n = 2). Gill tissues were rinsed with Dulbecco's phosphate‐buffered saline (DPBS), minced into 1–2 mm^3^ fragments, and enzymatically digested with collagenase II (2 mg/mL; Sigma‐Aldrich, St. Louis, MO, USA) and dispase (0.2 mg/mL; Roche, Basel, Switzerland) at 37°C for 15 min. Hemolymph was collected from the heart using a 1 mL syringe and immediately mixed 1:1 with EDTA anticoagulant to prevent coagulation. The resulting cell suspensions were filtered through a 40 µm cell strainer (Corning, Corning, NY, USA), followed by centrifugation at 300 × g for 4 min at 4°C. The supernatant was discarded, and the cell pellet was washed twice with DPBS containing 0.04% bovine serum albumin (BSA; Sigma‐Aldrich) and resuspended in DPBS. Cell viability was assessed using 0.2% trypan blue staining (Thermo Fisher Scientific, Waltham, MA, USA), and only samples with viability ≥85% and a cell concentration >1 × 10^6^ cells/mL were used for subsequent analyses.

### 5.7 ScRNA‐Seq Library Preparation and Sequencing

Single‐cell libraries were prepared using the Chromium Single Cell 3′ platform (10x Genomics, Pleasanton, CA, USA) following the manufacturer's protocol [77]. Briefly, single‐cell suspensions were loaded onto a microfluidic chip and partitioned with barcoded gel beads and reverse transcription reagents to generate Gel Bead‐In‐Emulsions (GEMs). After cell lysis, released mRNA was captured by oligo‐dT–containing barcoded beads and reverse transcribed to synthesize cDNA. The resulting cDNA was subsequently amplified by PCR to construct full‐length cDNA libraries. Library concentration was quantified using a Qubit 3.0 Fluorometer (Thermo Fisher Scientific) and quality was assessed using an Agilent 2100 Bioanalyzer (Agilent Technologies, Santa Clara, CA, USA), with libraries meeting an effective concentration threshold of >2 nM selected for sequencing. Paired‐end sequencing (PE150) was performed on an Illumina platform (Illumina, San Diego, CA, USA).

### 5.8 Preprocessing and Quality Control of scRNA‐seq Data

Raw sequencing data were aligned to the reference genome of *M. hainanense* [31] using Cell Ranger software (v7.2.0; 10x Genomics). Gene expression matrices were generated and subjected to stringent quality‐control filtering to remove low‐quality cells and potential doublets. Cells with fewer than 500 or more than 5,000 detected genes, as well as those with mitochondrial transcript content exceeding 20%, were excluded [78, 79, 80]. Putative doublets were further identified and removed using DoubletFinder [81]. These procedures ensured high data quality and enhanced the reliability of downstream analyses.

### 5.9 Cell Clustering and Cell Type Annotation

PCA was performed for dimensionality reduction, and cell clusters were identified using the shared nearest‐neighbor graph‐based clustering algorithm implemented in Seurat (v4.4.0; Satija Lab, New York, NY, USA) [82]. Clustered cells were visualized in two‐dimensional space using UMAP and t‐SNE. Differentially expressed genes among clusters were identified using the Wilcoxon rank‐sum test with thresholds of |log_2_FC| > 0.25 and adjusted *P* < 0.05. Cell type annotation was performed based on known and previously reported marker genes in aquatic and crustacean species, followed by manual curation to ensure annotation accuracy.

### 5.10 Cell–Cell Communication Analysis

Cell–cell communication analysis was performed using the CellChat R package (v2.1.0). Expressed genes from *M. hainanense* were mapped to orthologous genes in the CellChat ligand–receptor database, and potential ligand–receptor interactions among annotated gill cell populations were predicted based on their expression profiles. Communication probability was inferred from the expression of ligand–receptor pairs in sender and receiver cell populations and visualized as a heatmap. Representative ligand–receptor pairs were subjected to rigid‐body protein–protein docking using GRAMM. The top‐ranked complexes were visualized in PyMOL, and interface residues and intermolecular contacts were analyzed within 5 Å of the binding interface.

### 5.11 Gene Set Scoring

To evaluate the activity of signature gene sets in key cell populations under alkalinity stress, AUCell [83] was used to assess gene set enrichment at the single‐cell level. Genes were first ranked within each cell according to their expression levels, and enrichment of predefined gene sets was quantified by calculating the area under the recovery curve (AUC), providing a robust measure of gene set activity across individual cells.

### 5.12 Pseudotime Analysis

To characterize differentiation trajectories of gill cells and hemocytes under alkalinity stress, single‐cell pseudotime analysis was performed using Monocle2 [84]. Differentially expressed genes identified from clustering analysis were used for dimensionality reduction and trajectory inference, enabling the reconstruction of dynamic cellular transitions along pseudotime.

### 5.13 Weighted Gene Co‐Expression Network Analysis

Weighted gene co‐expression network analysis was conducted in R using the WGCNA package [85]. Following gene filtering, a weighted co‐expression network was constructed by selecting an appropriate soft‐thresholding power. The adjacency matrix was then converted into a topological overlap matrix (TOM) to identify gene co‐expression modules. Module–trait relationships were evaluated using module eigengenes to identify modules significantly associated with alkalinity stress. Hub genes within these modules were determined based on module membership and gene significance. Functional enrichment analyses, including Gene Ontology (GO) and KEGG pathway analysis, were subsequently performed for genes within key modules, and network visualization was carried out using Cytoscape software [86].

### 5.14 Fluorescence in Situ Hybridization

Gene‐specific probes targeting *V‐ATPase*, *LASP1*, *AChe*, *LPHN2*, *VEGFR1*, *proPO*, and *GDH* were synthesized by Wuhan Servicebio Technology Co., Ltd. (Wuhan, China) (Table S4). Fluorescence in situ hybridization (FISH) was performed using the RNASweAMI Multiple Fluorescence Detection Kit (Servicebio) according to the manufacturer's instructions. Paraffin‐embedded sections of gill and hemolymph were deparaffinized, rehydrated, subjected to heat‐induced antigen retrieval, and digested with protease prior to probe hybridization. Nuclei were counterstained with DAPI, and fluorescence signals were visualized and imaged using a fluorescence microscope. DAPI was used solely as a nuclear counterstain to visualize cell nuclei and tissue morphology and was not included in quantitative analyses. Quantification was performed exclusively using the fluorescence signals of the target markers.

### 5.15 RNA Interference

Gene‐specific primers for 5’ and 3’ RACE amplification were designed using Primer Premier 6 software (Premier Biosoft, Palo Alto, CA, USA) (Table S3), and the SMARTer RACE 5’/3’ Kit (Cat. No. 634858; Takara Bio Inc., Shiga, Japan) was used for cloning *MhGDH*. Based on the *M. hainanense* genome, the 2‐kb sequence upstream of the annotated ATG start codon of *MhGDH* was extracted. Putative cis‐regulatory motifs and transcription factor‐binding sites were predicted using PlantCARE and JASPAR, respectively. Primers containing T7 promoter sequences were then designed based on the MhGDH coding sequence for dsRNA synthesis (Table S3). Double‐stranded RNA was synthesized using the T7 RiboMAX Express RNAi System (Cat. No. P1700; Promega, Madison, WI, USA), purified by phenol/chloroform extraction, and stored at −80°C until use. EGFP and 1× PBS were used as negative controls. For in vivo delivery, dsRNA–transfection complexes were prepared using Entranster‐in vivo (Cat. No. 18668‐11‐1; Engreen Biosystem, Beijing, China) at a ratio of 2 µg dsRNA per 1 µL reagent. Healthy individuals of similar size were selected and injected intramuscularly with dsGDH at a dose of 2 µg/g body weight, followed by a second injection at 48 h to maintain knockdown efficiency. Following initiation of alkalinity stress, mortality was recorded at 6, 12, 24, 48, and 96 h for both control and experimental groups.

### 5.16 Western Blotting

Tissue samples were homogenized in RIPA lysis buffer supplemented with 1% protease inhibitor cocktail (Beyotime, Shanghai, China). After centrifugation, the supernatant was collected, and protein concentration was determined using a BCA Protein Assay Kit (Beyotime). Equal amounts of protein were separated by SDS‐PAGE and transferred onto nitrocellulose membranes (Millipore, Billerica, MA, USA). The membrane was initially probed with the primary antibody against the target protein, then stripped to remove bound antibodies, reblocked, and reprobed with an anti‐β‐actin antibody as the loading control. Protein signals were detected using an enhanced chemiluminescence (ECL) substrate (Thermo Fisher Scientific) and visualized with a Tanon imaging system (Tanon Science & Technology Co., Ltd., Shanghai, China). Band intensities were quantified using ImageJ software (National Institutes of Health) and GraphPad Prism (v10.1; GraphPad Software, San Diego, CA, USA).

### 5.17 Edu Assay for Primary Cell Proliferation

Primary gill cells and hemocytes under alkalinity stress were incubated with 10 µM EdU working solution for 2 h using the BeyoClick EdU Cell Proliferation Kit with AF488 (Cat. No. C0071S; Beyotime) according to the manufacturer's instructions. After incubation, cells were fixed with 4% paraformaldehyde for 15 min and permeabilized with 0.1% Triton X‐100 for 1 min. EdU incorporation was detected via a click chemistry reaction with AF488. Nuclei were counterstained with DAPI, and EdU‐positive cells were visualized using a fluorescence microscope. EdU‐positive cells and total nuclei were quantified in randomly selected microscopic fields using ImageJ (National Institutes of Health). The EdU‐positive rate was calculated as the percentage of EdU‐positive cells relative to the total number of DAPI‐stained nuclei.

### 5.18 Flow Cytometry

To evaluate mitochondrial functional alterations following GDH knockdown under alkalinity stress, ΔΨm and intracellular ROS levels were assessed by flow cytometry. After treatment, cells from each group were collected, washed with PBS, and stained with JC‐1 dye (Beyotime) or a ROS fluorescent probe (Beyotime) according to the manufacturer's instructions. For JC‐1 flow cytometric analysis, JC‐1 monomer fluorescence (green) was detected in the BL1‐H channel, whereas JC‐1 aggregate fluorescence (red) was detected in the BL2‐H channel. Quadrant gates were defined using the corresponding control samples for gill cells and hemocytes and were applied consistently to all treatment groups within each cell type. Cells in Q2 exhibited high red fluorescence, indicating a high ΔΨm, whereas cells in Q3 displayed increased green fluorescence and reduced red fluorescence, indicating mitochondrial depolarization. The red‐to‐green fluorescence intensity ratio was used to evaluate ΔΨm. Intracellular ROS levels were quantified based on the fluorescence intensity of the ROS probe. All samples were analyzed using a flow cytometer (BD Biosciences, San Jose, CA, USA), and data were processed using FlowJo software (BD Biosciences).

### 5.19 Untargeted Metabolomics Analysis

To further characterize metabolic alterations induced by GDH knockdown under alkalinity stress, untargeted metabolomic profiling was performed to compare metabolite changes among experimental groups. Following treatment, samples were collected, and metabolites were extracted and subjected to liquid chromatography–mass spectrometry (LC–MS) analysis (Thermo Fisher Scientific). Raw data were processed and normalized prior to statistical analysis. PCA was conducted to assess sample distribution and clustering patterns among groups. To identify metabolites with significant intergroup differences, orthogonal partial least squares discriminant analysis (OPLS‐DA) was performed. Differential metabolites were screened based on the following criteria: variable importance in projection (VIP) ≥ 1.0, Student's *t*‐test *P* < 0.05, and |log_2_FC| ≥ 1. Identified metabolites were subsequently annotated and subjected to pathway enrichment analysis using the KEGG database. Key metabolites were visualized using heatmaps, and metabolic pathway maps were constructed to summarize the major metabolic changes associated with GDH knockdown under alkalinity stress.

### 5.20 Statistical Analysis

All data were presented as the mean ± standard deviation (SD) from at least three independent biological replicates. Statistical analyses were performed using IBM SPSS Statistics (v29.0.2; IBM Corp., Armonk, NY, USA). Data normality and homogeneity of variance were assessed using the Shapiro–Wilk and Levene's tests, respectively. Differences among multiple groups were analyzed by one‐way analysis of variance (ANOVA) followed by Tukey's post hoc test, whereas comparisons between two groups were performed using a two‐tailed Student's *t*‐test. Different lowercase letters indicate significant differences among multiple groups (*P* < 0.05). Exact *P* values for pairwise comparisons were shown in the figures.

## Author Contributions

**S.S**. and **Y.J**. conceived and designed the study. **Y.J**. and **Z.L**. performed cell type annotation. **C.B**., **N.Z**., and **J.Q**. conducted the bioinformatics analyses. **Y.J**., **Z.L**., and **Q.Y**. carried out experimental validation. **Y.J**. prepared the original draft of the manuscript. **S.S**., **C.B**., and **N.Z**. reviewed and edited the manuscript. **S.S**. supervised the study. **Y.J**. and **Z.L**. contributed to manuscript drafting with input from all authors.

## Conflicts of Interest

The authors declare no conflicts of interest.

## Supporting information




**Supporting File 1**: advs76978‐sup‐0001‐SuppMat.docx.

**Supporting File 2**: advs76978‐sup‐0002‐TableS1‐S6.xlsx.

## Data Availability

The data that support the findings of this study are openly available in National Genomics Data Center (NGDC) at https://ngdc.cncb.ac.cn/gsa/, reference number PRJCA062493.
