# Supplementary material for: Single‐Cell Transcriptomics and Metabolomics Reveal Glutamate Dehydrogenase as a Central Regulator of Nitrogen Metabolic Remodeling During Alkalinity Adaptation in Crustaceans
Source: Adv Sci (Weinh). 2026 Aug 5;13(60):e76978. doi: 10.1002/advs.76978 (PMC13440371; doi:10.1002/advs.76978)
Supplement: Supplementary file 1 [file ADVS-13-e76978-s002.docx]

**Single-Cell Transcriptomics and Metabolomics Reveal Glutamate Dehydrogenase as a Central Regulator of Nitrogen Metabolic Remodeling during Alkalinity Adaptation in Crustaceans**

Yiting Jin^a^, Zhimin Lv^a^, Chao Bian^b^, Qibin Yang^c^, Na Zhou^d^, Jianguang Qin^e^, Shengming Sun^a,^*

^a^Key Laboratory of Exploration and Utilization of Aquatic Genetic Resources, Ministry of Education, Shanghai Ocean University, Shanghai 201306, China

^b^Laboratory of Aquatic Genomics, College of Life Sciences and Oceanography, Shenzhen University, Shenzhen 518057, China

^c^Tropical Aquaculture Research and Development Center, South China Sea Fisheries Research Institute, Chinese Academy of Fishery Sciences, Sanya 572018, China

^d^School of Pharmacy, Laboratory of Drug Discovery from Natural Resources and Industrialization, State Key Laboratory of Mechanism and Quality of Chinese Medicine, Macau University of Science and Technology, Taipa, Macau, China

^e^College of Science and Engineering, Flinders University, Adelaide 5042, Australia

* Corresponding author at: Key Laboratory of Exploration and Utilization of Aquatic Genetic Resources, Ministry of Education, Shanghai Ocean University, Shanghai 201306, China

E-mail address: sunshengming621416@163.com (S.M. Sun).

**Supporting Information**

**Figure S1. Effects of alkalinity stress on urea content in different tissues and ammonia concentration in culture water.**

**Figure S2. Cell clustering and annotation of gill cells and hemocytes based on single-cell transcriptomic analysis.**

**Figure S3. Predicted cell–cell communication and molecular docking of representative ligand–receptor pairs.**

**Figure S4. KEGG pathway enrichment analysis of differentially expressed genes in major gill and hemocyte cell types under alkalinity stress.**

**Figure S5. Cell type-specific gene set scores in gill and hemocyte populations under control and alkalinity stress conditions.**

**Figure S6. Module–module relationship analysis based on weighted gene co-expression network analysis (WGCNA).**

**Figure S7. Expression patterns of genes in the blue module and the corresponding module eigengene profile.**

**Figure S8. Distribution of transcription factors among different WGCNA modules.**

**Figure S9. Molecular characterization and structural comparison of the *GDH* gene from *M. hainanense*.**

**Figure S10. Phylogenetic analysis of *MhGDH*.**

**Figure S11. Prediction of cis-regulatory elements and a putative USF2-binding site in the *MhGDH* promoter.**

**Figure S12. Tissue distribution of nitrogen metabolism-related genes.**

**Figure S13. Pathway enrichment analysis of differential metabolites following GDH knockdown under alkalinity stress.**

**Figure S1. Effects of alkalinity stress on urea content in different tissues** **and ammonia concentration in culture water.**

A) Urea content in hemocytes following exposure to alkalinity stress for 0, 6, 12, 24, 48, and 96 h. B) Urea content in the gills and antennal glands at the corresponding time points under alkalinity stress. C) Ammonia concentration in culture water following exposure to alkalinity stress for 0, 6, 12, 24, 48, and 96 h. The CK group consisted of the same experimental water maintained under identical conditions but without prawns and served as an animal-free water control. Data are presented as mean ± SD. Statistical significance was determined by one-way analysis of variance (ANOVA) followed by Duncan's multiple range test. Different lowercase letters indicate significant differences among time points within each tissue (*P* < 0.05).

**Figure S2. Cell clustering and annotation of gill cells and hemocytes based on single-cell transcriptomic analysis.**

A) t-Distributed stochastic neighbor embedding (t-SNE) visualization of gill cells. The left panel displays the original cell clusters, and the right panel shows the corresponding cell-type annotations, including pillar cells, nephrocytes, endothelial cells, monocytes, mucous cells, septal cells, pericytes, chloride cells, neuroepithelial cells, and undefined cells. B) t-SNE visualization of hemocytes. The left panel displays the original cell clusters, and the right panel shows the corresponding cell-type annotations, including precursor hemocytes, differentiating hemocytes, semi-granulocytes, granulocytes, specific granule-containing hemocytes, and undefined cells.

**Figure S3. Predicted cell–cell communication and molecular docking of representative ligand–receptor pairs.**

A) Rows represent sender cell types, and columns represent receiver cell types. Color intensity indicates the relative communication score, with darker colors representing stronger predicted interactions. B) Molecular docking models of representative ligand–receptor pairs involved in communication with hemocytes. The upper panel shows the interaction between pericyte-derived RGMB and hemocyte-expressed NEO1, whereas the lower panel shows the interaction between endothelial cell-derived IL1RAPL1 and hemocyte-expressed PTPRF. Ligands are shown in yellow and receptors in blue. Enlarged views show the predicted binding interfaces and key interacting residues.

**Figure S4. KEGG pathway enrichment analysis of differentially expressed genes in major gill and hemocyte cell types under alkalinity stress.**

A) KEGG pathway enrichment analysis of differentially expressed genes in major gill cell types, including pillar cells, nephrocytes, endothelial cells, septal cells, hemocytes, and neuroepithelial cells, under alkalinity stress. B) KEGG pathway enrichment analysis of differentially expressed genes in major hemocyte cell types, including precursor hemocytes and semi-granulocytes, under alkalinity stress. The x-axis indicates the number of genes, and the color scale represents enrichment significance as –log_10_(*P* value).

**Figure S5. Cell type-specific gene set scores in gill and hemocyte populations under control and alkalinity stress conditions.**

A) Boxplots showing the distributions of gene set scores for the ABC transporter pathway (left) and collecting duct acid secretion (right) across major gill cell populations under control and alkalinity stress conditions. B) Boxplots showing the distributions of gene set scores for the phagosome pathway (left) and Toll-like receptor signaling pathway (right) across hemocyte subpopulations under control and alkalinity stress conditions.

**Figure S6. Module–module relationship analysis based on weighted gene co-expression network analysis (WGCNA).**

The heatmap shows the correlations among co-expression modules identified by WGCNA. Rows and columns represent individual modules, and the color scale indicates the strength and direction of the correlations. The values within each cell represent the correlation coefficient, with the corresponding *P* value shown in parentheses.

**Figure S7. Expression patterns of genes in the blue module and the corresponding module eigengene profile.**

The upper panel shows a heatmap of gene expression within the blue module across different samples, with red and green indicating relatively high and low expression levels, respectively. The lower panel shows the corresponding module eigengene values across the same samples, reflecting the overall expression pattern of the blue module.

**Figure S8. Distribution of transcription factors among different WGCNA modules**.

The bar chart shows the number of transcription factors (TFs) identified in each co-expression module. The salmon module contained the largest number of TFs, followed by the blue and cyan modules, whereas the violet and darkorange modules contained relatively few TFs.

**Figure S9. Molecular characterization and structural comparison of the *GDH* gene from *M. hainanense*.**

A) Schematic representation of the full-length *GDH* cDNA, showing the 5′ untranslated region (gray), the open reading frame (orange), and the 3′ untranslated region. B) Nucleotide and deduced amino acid sequences of MhGDH. The initiation codon (ATG) and termination codon (TAA) are highlighted in red, and the deduced amino acid sequence is shown below the corresponding nucleotide sequence. C) Predicted three-dimensional structure of MhGDH protein and structural comparison with homologous proteins from *M. nipponense*, *Scylla paramamosain*, *Scophthalmus maximus*, *Drosophila grimshawi*, and *Theropithecus gelada*.

**Figure S10. Phylogenetic analysis of *MhGDH*.**

*M. hainanense* is highlighted in red and marked with a triangle. Circle size at each node represents bootstrap support, as indicated in the legend, with larger circles corresponding to higher support values.

**Figure S11. Prediction of cis-regulatory elements and a putative USF2-binding site in the *MhGDH* promoter.**

A) Distribution of predicted cis-regulatory elements and transcription factor-binding sites within the 2-kb region upstream of the annotated translation start site of *MhGDH*. Selected cis-regulatory elements and stress-related transcription factor-binding sites are indicated above the sequence. The predicted USF2-binding site is highlighted by a red box. B) Location and motif profile of the putative USF2-binding site in the *MhGDH* promoter. The site spans -1178 to -1168 bp relative to the annotated translation start site and corresponds to the sequence 5′-GGCAGGTGATA-3′ in the upstream region of *MhGDH*. The sequence logo and position frequency matrix illustrate the nucleotide preferences of the USF2 motif used in the prediction.

**Figure S12. Tissue distribution of nitrogen metabolism-related genes.**

A) Representative RT-PCR analysis showing the expression patterns of *GDH*, *Rhag*, *GS*, and *GOGAT* across eight tissues, including the brain, eye, antennal gland, heart, gill, hepatopancreas, muscle, and intestine. *β-actin* was used as the internal reference gene. B) Heatmap showing the relative expression levels of *GDH*, *Rhag*, *GS*, and *GOGAT* across different tissues. Data from three biological replicates were normalized to the brain and are presented as relative fold changes.

**Figure S13. Pathway enrichment analysis of differential metabolites following GDH knockdown under alkalinity stress.**

A) KEGG enrichment bubble plot of differential metabolites between the PBS and dsEGFP-Alkalinity groups. B) KEGG enrichment bubble plot of differential metabolites between the PBS and dsGDH-Alkalinity groups. C) KEGG enrichment bubble plot of differential metabolites between the dsEGFP-Alkalinity and dsGDH-Alkalinity groups. The x-axis represents the rich factor, and the y-axis shows the significantly enriched metabolic pathways. Bubble size indicates the number of differential metabolites mapped to each pathway, whereas bubble color represents the enrichment significance (*P* value).
